# Targeting Nuclear NAD^+^ Synthesis Inhibits DNA Repair, Impairs Metabolic Adaptation and Increases Chemosensitivity of U-2OS Osteosarcoma Cells

**DOI:** 10.3390/cancers12051180

**Published:** 2020-05-07

**Authors:** Alexandra Kiss, Arnold Péter Ráduly, Zsolt Regdon, Zsuzsanna Polgár, Szabolcs Tarapcsák, Isotta Sturniolo, Tarek El-Hamoly, László Virág, Csaba Hegedűs

**Affiliations:** 1 Department of Medical Chemistry, Faculty of Medicine, University of Debrecen, H-4032 Debrecen, Hungary; kissalexandra@med.unideb.hu (A.K.); radulyarnold27@gmail.com (A.P.R.); regdon.zsolt@med.unideb.hu (Z.R.); polgar.zsuzsanna@med.unideb.hu (Z.P.); isotta.sturniolo@med.unideb.hu (I.S.); tahamoly@hotmail.com (T.E.-H.); 2 Doctoral School of Molecular Medicine, University of Debrecen, H-4032 Debrecen, Hungary; 3 Department of Biophysics and Cell Biology, Faculty of Medicine, University of Debrecen, H-4032 Debrecen, Hungary; tarapcsakszabolcs@gmail.com; 4 Drug Radiation Research Department, National Center for Radiation Research and Technology, Atomic Energy Authority, Cairo 113701, Egypt; 5 MTA-DE Cell Biology and Signaling Research Group, H-4032 Debrecen, Hungary

**Keywords:** NAD^+^, NMNAT1, cisplatin, chemotherapy, apoptosis, PARP1, osteosarcoma, cancer

## Abstract

Osteosarcoma (OS) is the most common bone tumor in children and adolescents. Modern OS treatment, based on the combination of neoadjuvant chemotherapy (cisplatin + doxorubicin + methotrexate) with subsequent surgical removal of the primary tumor and metastases, has dramatically improved overall survival of OS patients. However, further research is needed to identify new therapeutic targets. Here we report that expression level of the nuclear NAD synthesis enzyme, nicotinamide mononucleotide adenylyltransferase-1 (NMNAT1), increases in U-2OS cells upon exposure to DNA damaging agents, suggesting the involvement of the enzyme in the DNA damage response. Moreover, genetic inactivation of NMNAT1 sensitizes U-2OS osteosarcoma cells to cisplatin, doxorubicin, or a combination of these two treatments. Increased cisplatin-induced cell death of NMNAT1^−/−^ cells showed features of both apoptosis and necroptosis, as indicated by the protective effect of the caspase-3 inhibitor z-DEVD-FMK and the necroptosis inhibitor necrostatin-1. Activation of the DNA damage sensor enzyme poly(ADP-ribose) polymerase 1 (PARP1), a major consumer of NAD^+^ in the nucleus, was fully blocked by NMNAT1 inactivation, leading to increased DNA damage (phospho-H2AX foci). The PARP inhibitor, olaparib, sensitized wild type but not NMNAT1^−/−^ cells to cisplatin-induced anti-clonogenic effects, suggesting that impaired PARP1 activity is important for chemosensitization. Cisplatin-induced cell death of NMNAT1^−/−^ cells was also characterized by a marked drop in cellular ATP levels and impaired mitochondrial respiratory reserve capacity, highlighting the central role of compromised cellular bioenergetics in chemosensitization by NMNAT1 inactivation. Moreover, NMNAT1 cells also displayed markedly higher sensitivity to cisplatin when grown as spheroids in 3D culture. In summary, our work provides the first evidence that NMNAT1 is a promising therapeutic target for osteosarcoma and possibly other tumors as well.

## 1. Introduction

Bone tumors constitute 4–7% of all cancers in children aged 0–14 years and 7–8% in children aged 15–19 years [1]. Osteosarcoma is the most prevalent neoplasm of the bone in children and adolescents, with approximately 850,000 cases reported each year in the US. Tumors are typically diagnosed between 10 and 30 years of age and mostly arise in the femur, tibia, and humerus. Although most osteosarcoma cases are sporadic, a higher incidence of osteosarcoma has been reported in certain hereditary cancer syndromes. For example, mutations of the tumor suppressor retinoblastoma protein (Rb) in retinoblastoma [2] or p53 in Li-Fraumeni syndrome [3], as well as the DNA repair helicase Werner protein are also associated with osteosarcoma [4]. Previous radiotherapy of other tumors may also lead, in the long term, to osteosarcoma development.

Treatment of osteosarcoma usually begins with neoadjuvant chemotherapy followed by surgical removal of the primary tumor and metastases (typically localized to the lungs). Agents used for osteosarcoma chemotherapy include cisplatin, methotrexate, and doxorubicin [5]. In the European Union, the liposome-encapsulated synthetic muramyl dipeptide analog, mifamurtide, is also approved by the European Medicines Agency for the immunotherapy of osteosarcoma [6]. The five-year survival rate depends on the stage, type, and subtype of osteosarcoma and ranges from 60% to 75% [7]. The rather poor therapeutic responsiveness indicates that new treatment modalities are clearly needed to improve the disease-free survival of osteosarcoma patients.

Neoplastic transformation is accompanied by fundamental rearrangements of metabolic pathways. A key hallmark of cancer metabolism is the reliance of tumor cells on glycolysis, even if oxygen is available (aerobic glycolysis, also known as the Warburg effect) [8]. NAD^+^ is a central metabolite of energy production that serves as an electron carrier in glycolysis and the tricarboxylic acid (TCA) cycle. In addition to its role as a metabolic cofactor, NAD^+^ also has signaling roles [9]. NAD^+^-derived signaling messengers include the calcium-mobilizing agents cyclic ADP-ribose [10] and nicotinic acid adenine dinucleotide phosphate (NAADP) [11]. Moreover, members of the ADP-ribosyltransferase (ART) and sirtuin enzyme families also use NAD^+^ as a substrate converting it to ADP-ribose and nicotinamide [12]. Sirtuins are NAD^+^-dependent histone deacetylases that transfer the removed acetyl group onto NAD^+^-derived ADP-ribose to generate O-acetyl-ADP-ribose, whereas ART enzymes cleave NAD^+^ to nicotinamide and ADP-ribose and transfer the latter onto protein acceptors [13]. Some ARTs function as mono-ADP-ribosyltransferases while others cleave multiple NAD^+^s and polymerize the resulting ADP-ribose moieties onto suitable protein acceptors, resulting in protein poly(ADP-ribosyl)ation (PARylation) [14,15]. This latter group includes poly(ADP-ribose) polymerase-1 (PARP1; also known as ARTD1), PARP2 (ARTD2), and tankyrases (ARTD5-6) [16]. PARP1, a major NAD^+^ consumer, plays a central role in tumor biology [17]. On one hand, PARP1 regulates DNA replication, gene expression, DNA repair, cell adhesion, and migration [18]. On the other hand, PARP1 is the primary target for a novel cancer therapeutic modality based on the synthetic lethality paradigm [19,20]. Clinically used PARP inhibitors (PARPi) take advantage of the vulnerability of cancer cells (e.g., ovarian and breast carcinomas) that carry mutations in the DNA repair genes BRCA1/2, rendering them sensitive to DNA repair inhibitors, such as PARPi compounds. PARP inhibitors have also been evaluated as chemosensitizers (e.g., in combination with the DNA alkylating agent temozolomide or the topoisomerase inhibitor topotecan) [21,22,23], but myelotoxicity prevented the successful translation of promising preclinical results into the clinic.

The question arises whether and how NAD^+^ metabolism can be targeted for cancer therapy. NAD^+^ is synthesized through multiple routes [24]. In humans, the central pathway relies on nucleobases with special preference for nicotinamide (Nam), although nicotinic acid (NA) can also serve as starting material for NAD^+^ synthesis [25]. Nicotinamide phosphoribosyltransferase (NAMPT) enzymes can use Nam and phosphoribosyl pyrophosphate (PRPP) to make nicotinamide mononucleotide (NMN) [26]. In turn, nicotinamide mononucleotide adenosyltransferases (NMNAT) catalyze the final step by forming NAD^+^ from NMN and ATP. Tissue and subcellular distribution of the three NMNAT isoforms (NMNAT1-3) show distinct patterns [27]. Besides the nuclear (NMNAT1) and cytoplasmic/Golgi (NMNAT2) isoforms, a mitochondrial NAD^+^ synthase (NMNAT3) has also been described; however, the mitochondrial localization and contribution of NMNAT3 to the mitochondrial NAD^+^ pool has been questioned [28]. Nuclear NAD^+^ synthesis is of special interest because several major NAD^+^-consuming enzymes (PARP1,2, SIRT1,6,7) that mediate important survival mechanisms operate in the nuclear compartment [29].

Since inhibitor molecules are available for NAMPT, our knowledge on the targetability of NAD^+^ synthesis for cancer therapy comes from experiments with the NAMPT inhibitor compounds, FK866 and GMX1777. Although these drug candidates efficiently kill cancer cells, they also show some toxicity in non-transformed cells. In clinical trials, these drugs were relatively well-tolerated and displayed acceptable safety profiles [30]. 2,3-Dibromo-1,4-naphthoquinone (DBNQ) was recently identified as an inhibitor of purified NMNAT-1 [31]. However, this finding needs to be confirmed in cell-based assays.

The role of NMNAT1 in cancer cells is not known and its role in cancer cell chemosensitivity has not yet been investigated. In our current study, we aimed to investigate whether NMNAT1 plays a role in maintaining cell viability and whether it contributes to osteosarcoma cell survival following genotoxic stimuli. We report that U-2OS human osteosarcoma cells tolerate the genetic inactivation of the NMNAT1 gene and show increased susceptibility to cisplatin and doxorubicin. Increased chemosensitivity of the NMNAT1^−/−^ phenotype is likely due in part to restricted DNA-damage-induced protein PARylation and consequently enhanced DNA damage. U-2OS cells unable to respond sufficiently to cisplatin treatment undergo caspase-mediated apoptotic and caspase-independent necroptotic death, suggesting that NMNAT1 may be worth investigating further as a potential target in cancer therapy with special regard to osteosarcoma treatment.

## 2. Results

### 2.1. DNA Damaging Drugs Induce NMNAT1 Expression in Tumor Cell Lines

**Figure 1:**
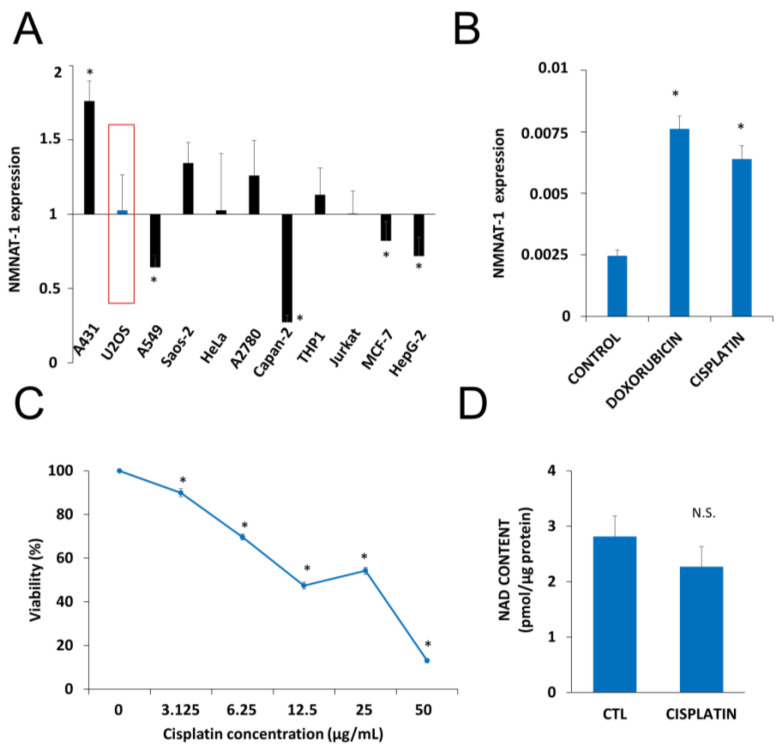
Nicotinamide mononucleotide adenylyltransferase-1 (NMNAT1) mRNA expression and induction by antitumor agents. The expression of NMNAT1 mRNA was determined in eleven human tumor cell lines. The y-axis shows the average expression level of the tested cell lines. Bars marked with asterisks are significantly different from the average expression (Student–Newman–Keuls method; * *p* < 0.05) (**A**). NMNAT1 expression in the U-2OS cell line was induced 24 h after cisplatin (6.25 μg/mL) or doxorubicin (2 μg/mL) treatment. Bars marked with asterisks are significantly different from the control (Dunnett test; * *p* < 0.05) (**B**). Calcein acetoxymethyl (Calcein AM) assay, indicating the concentration-dependent cytotoxic effect of cisplatin (3.125–50 μg/mL) on U-2OS cells, was measured 24 h after cisplatin treatment. Bars marked with asterisks are significantly different from the control (Bonferroni test; * *p* < 0.05) (**C**). Total NAD^+^ content was measured in cell lysates 24 h after cisplatin (6.25 μg/mL) treatment and normalized to protein content. Bars marked with asterisks are significantly different from the control (Student’s *t* test; * *p* < 0.05, N.S.: not significant) (**D**). Data plotted are means ± SEM (*n* = 3).

Up to date NMNAT1 mRNA expression levels for osteosarcoma cells (U-2OS, SAOS-2) were not available. That is why we checked the mRNA levels in osteosarcoma cells and compared with other human tumor cell lines of different origins. Eleven human tumor cell lines were tested for NMNAT-1 mRNA expression (Figure 1A). The transcript could be detected, although at different levels, in all cell lines. Compared to the average expression level, A431 cells displayed significantly higher mRNA expression, while significantly lower expression was detected in A549, Capan2, MCF7 and HepG2 cell lines (Figure 1A). U-2OS osteosarcoma cells which showed average levels of NMNAT1 expression were chosen for further investigation. Of the three human NMNAT isoforms, NMNAT-1 is most highly expressed, in U-2OS cells, while NMNAT2 and NMNAT-3 are expressed at lower levels (Figure S1A). The DNA damaging chemotherapeutic agents doxorubicin and cisplatin significantly upregulated NMNAT1 mRNA expression (Figure 1B), suggesting that the enzyme may be a survival factor in DNA damage situations. Cisplatin, one of the drugs most commonly used for the treatment of osteosarcoma, caused a marked elevation in NMNAT1 protein expression in U-2OS cells (Figure S1B). Cisplatin treatment caused a concentration-dependent cytotoxicity in U-2OS cells (Figure 1C). Based on the viability data, we chose the 6.25 µg/mL concentration for the following experiments. At this concentration, cisplatin caused no significant change in NAD^+^ levels (Figure 1D).

### 2.2. Generation and Characterization of an NMNAT1^−/−^ Cell Line

**Figure 2:**
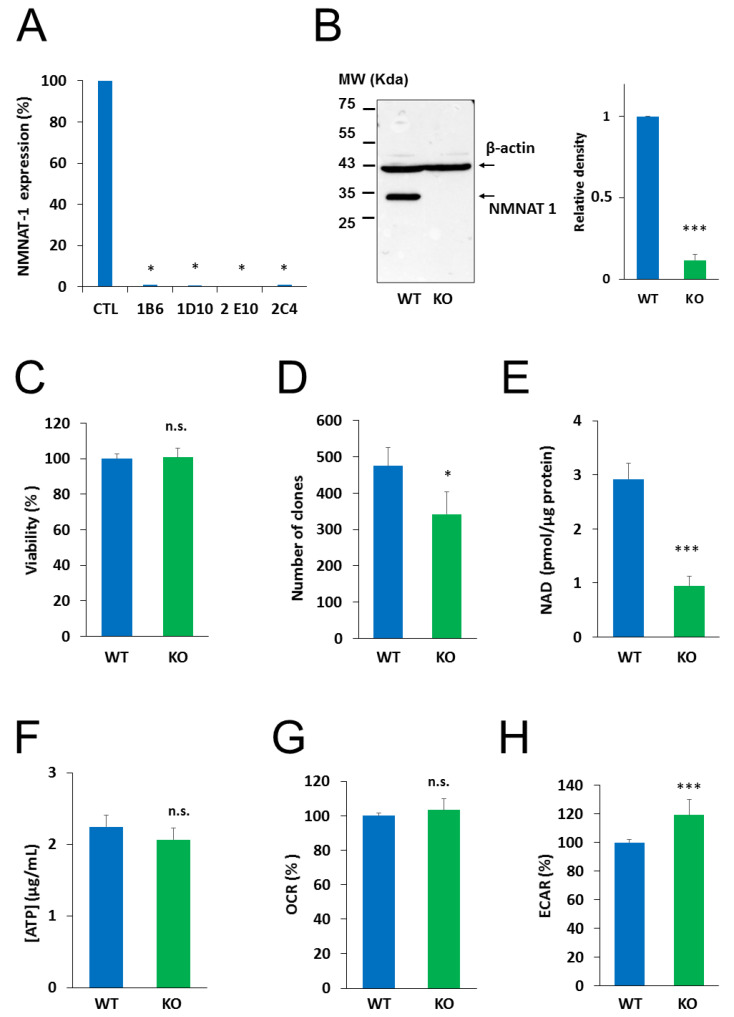
Characterization of NMNAT 1 KO cell line. NMNAT1 knockout cell lines were generated with CRISPR-CAS9 technology. Puromycin resistant cells were sorted and single cell colonies were grown. NMNAT1 mRNA levels were measured with RT-QPCR in each colony. Results are expressed as a percentage of NMNAT1 expression of the wild type U-2OS cell line (control). Bars marked with asterisks are significantly different from the control (Dunnett test; * *p* < 0.05) (**A**). Clone 1B6 was chosen for further investigation. NMNAT1 protein was measured in cell lysates of wild type U-2OS and the 1B6 clone with Western blot (**B**). Full WB image can be found in Supplementary Material. The following experiments compare the basic characteristics of wild type (WT) and NMNAT1 knockout (KO) cells. Viability was measured with a Calcein AM viability assay. Data points marked with asterisks are significantly different from the control (Student’s t test; * *p* < 0.05, N.S.: not significant) (**C**). Clonogenic activity was assessed on day 6 by counting crystal violet stained colonies. Bars marked with asterisks are significantly different from the control (Student’s *t* test; * *p* < 0.05, N.S.: not significant) (**D**). Basal total NAD^+^ (**E**) and ATP (**F**) levels were assayed from cell lysates and normalized to protein content. Bars marked with asterisks are significantly different from the control (Student’s *t* test; *** *p* < 0.001, N.S.: not significant) Metabolic parameters such as oxygen consumption rate (OCR)/oxidative phosphorylation (**G**) and extracellular acidification rate (ECAR)/glycolytic activity (**H**) were measured with a Seahorse metabolic analyzer. Bars marked with asterisks are significantly different from the control (Student’s *t* test; *** *p* < 0.001, n.s.: not significant). Data plotted are means ± SEM (*n* = 3).

To investigate the role of NMNAT1 in the survival of cisplatin-treated cells, we inactivated the gene for NMNAT1 using CRISPR-Cas9 technology. Single cell clones were obtained by cell sorting from cultures of NMNAT1^−/−^ cells. We tested all the clones and all of them lacked NMNAT1 mRNA (Figure 2A). Clone 1B6 was selected for downstream experiments. Western blotting proved that NMNAT1 protein was missing from this clone (Figure 2B). Morphological properties of wild type and NMNAT1^−/−^ cells (Figure S2A) revealed a significant reduction in the nuclear size and cell size (Figure S2B and C). The nuclear and cellular roundness was also slightly but significantly affected by the absence of a functional NMNAT1 protein (Figure S2D,E). The NMNAT1 deficient U-2OS cell line showed unaltered cell viability, as determined using the Calcein acetoxymethyl (Calcein AM) method (Figure 2C). However, clonogenic activity was impaired in the absence of a functional enzyme (Figure 2D). Despite elevated NMNAT-2 expression (Figure S1A), total cellular NAD^+^ levels dropped to approximately one third of the control cell line (Figure 2E), indicating that NMNAT1 plays a dominant role in cellular NAD^+^ synthesis. Interestingly, lower NAD^+^ levels in NMNAT1^−/−^ cells did not suppress ATP levels (Figure 2F) or impair cellular respiration, as indicated by the unchanged oxygen consumption rate (Figure 2G). Extracellular acidification rate (ECAR), a measure of glycolysis, showed higher values in the absence of NMNAT1 compared to the parent cell line (Figure 2H).

### 2.3. NMNAT-1 Inactivation Increases Chemosensitivity of the U-2OS Osteosarcoma Cells

**Figure 3:**
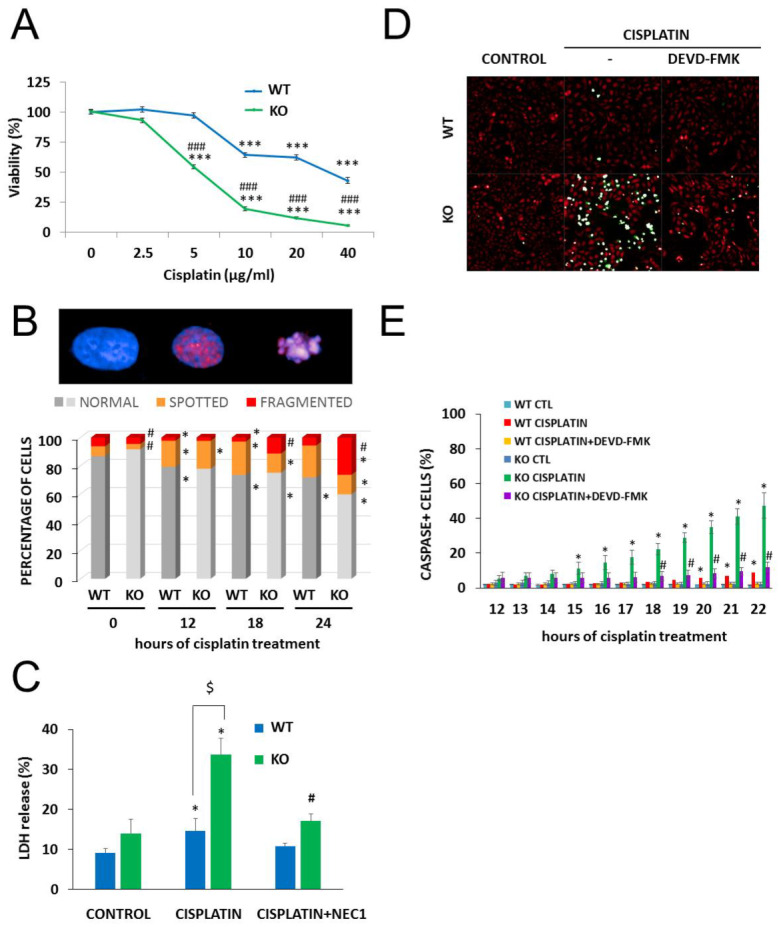
Chemosensitivity of NMNAT-1 KO cell line. Chemosensitivity of wild type (WT) and knockout (KO) cells was compared in a Calcein-AM viability assay 24 h after treatment with different concentrations of cisplatin (0.625–10 μg/mL). A new batch of cisplatin was used for this and all subsequent experiments. Results are expressed as percentages, compared to the vehicle (medium) treated samples. Data points marked with asterisks are significantly different from the control (Bonferroni test; *** *p* < 0.001). Data points marked with hashmarks are significantly different from the corresponding treatment of the wild type cells (Bonferroni test; ### *p* < 0.001). (**A**). Cisplatin-induced DNA damage was assessed by quantifying the P-H2AX signal after immunofluorescent staining. High-content analysis detected three different cell morphologies, “normal”, “spotted” and “fragmented”, representative images are shown (**B**). Chart shows the percentage of the three types of morphologies in untreated and in cisplatin-treated samples at 12, 18, and 24 h after cisplatin treatment. Bars marked with asterisks are significantly different from the control (0 hours of cisplatin treatment) (Student–Newman–Keuls method; * *p* < 0.05). Bars marked with hashmarks are significantly different from the corresponding treatment of the wild type cells (Student–Newman–Keuls method; # *p* < 0.05) (**B**). Error bars are not shown on panel B because of the 3D presentation. Lactate dehydrogenase (LDH) release was determined from the supernatants at 24 h after cisplatin treatment and expressed as a percentage of the positive control (lysed cells). Cells were treated with cisplatin or with the combination of cisplatin with the necroptosis inhibitor, concentration necrostatin-1 (NEC1). Bars marked with asterisks are significantly different from the control (not shown) (Dunnett test; * *p* < 0.05). Bars marked with hashmarks are significantly different from the cisplatin-treated samples (Dunnett test; # *p* < 0.05). Bars marked with $ are significantly different from the corresponding treatment of the wild type cells (Dunnett test; $ *p* < 0.05) (**C**). Apoptotic cell death was measured with a fluorogenic caspase-3 substrate in a kinetic assay on a Perkin Elmer Opera Phenix High Content Analyzer. Representative images (**D**) show the caspase activity signal (green) with digital phase contrast (red) at 22 h after cisplatin treatment. Images were taken every hour between 12 and 22 h after cisplatin treatment. Some samples were pretreated with the caspase-3 inhibitor z-DEVD-FMK as shown. The numbers of caspase positive cells are shown as a percentage of the actual number of cells. Data points marked with asterisks are significantly different from the control (12 h of cisplatin treatment) (Student–Newman–Keuls method; * *p* < 0.05). Data points marked with hashmarks are significantly different from the cisplatin treated samples Student–Newman–Keuls method; # *p* < 0.05). (**E**). Means ± SEM are shown (*n* = 3).

Next, we investigated the role of NMNAT1 in the survival/death of cisplatin-treated cells. We found that the absence of NMNAT1 sensitized cells to the toxic effects of cisplatin (Figure 3A). For example, 5 µg/mL cisplatin did not cause significant toxicity in the parent cell line but killed 45% of NMNAT1^−/−^ cells (Figure 3A). Morphological phenotyping of cisplatin-treated cells was performed to understand the mechanism of chemosensitization in NMNAT1^−/−^ cells.

Cisplatin is a DNA crosslinking agent that does not induce DNA breaks directly. γH2AX foci, indicating DNA double strand breaks (DSB), have been reported in cisplatin-treated cells and reflect activation of DNA repair mechanisms, such as nucleotide excision repair or non-homologs end joining [32]. High-content analysis detected three different cell morphologies: “normal” cells contained no P-H2AX signal, “spotted” cell type contained P-H2AX foci, while the “fragmented” type showed a condensed morphology, with a diffuse P-H2AX signal. We measured γH2AX and analyzed staining in combination with cell fragmentation, a marker of cell death that indicates failed DNA repair. In line with previous reports [32], cisplatin caused H2AX phosphorylation (γH2AX formation) in wild type cells. Significantly more cells were positive for γH2AX formation and/or showed signs of nuclear fragmentation in cisplatin-treated NMNAT1 deficient cells compared to their wild type counterparts (Figure 3B).

Cisplatin-induced cell death occurred via multiple pathways in the absence of NMNAT1. Loss of plasma membrane integrity, a sign of necroptosis, was observed, as indicated by increased lactate dehydrogenase (LDH) release in cisplatin-treated NMNAT-1^−/−^ cells. Necrostatin-1 (NEC1) abolished LDH release, suggesting that it was indeed the consequence of necroptosis (Figure 3C). The involvement of the caspase-mediated apoptotic cell death pathway was also clearly demonstrated with a fluorogenic caspase-3/7 substrate and inhibition of the signal by z-DEVD-FMK (Figure 3D,E).

**Figure 4:**
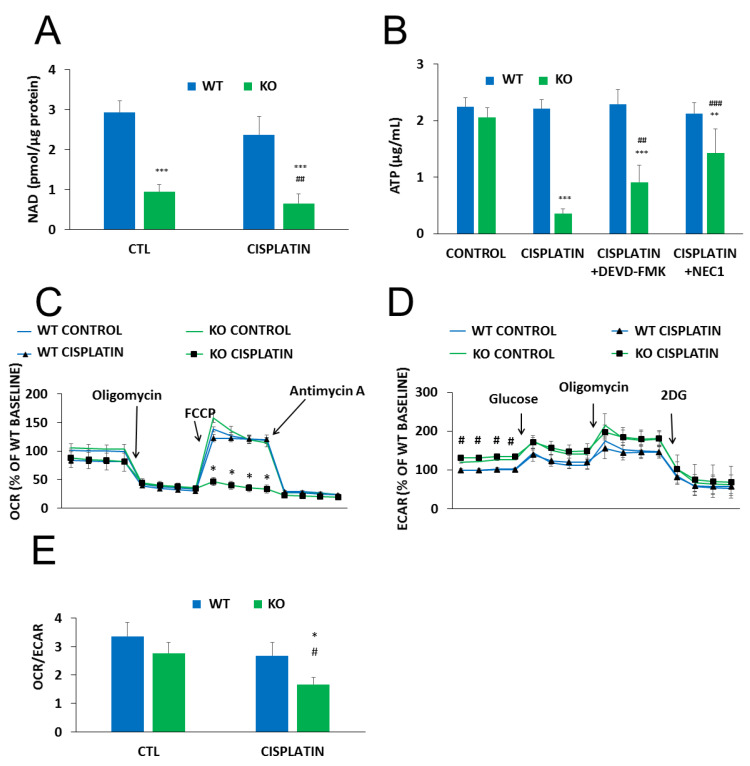
Metabolic alterations in NMNAT-1 KO cell line. Following cisplatin (5 μg/mL) treatment (24 h), total NAD^+^ levels were determined from cell lysates of WT and KO cells and normalized to protein content. Bars marked with asterisks are significantly different from the control (Bonferroni test; *** *p* < 0.001). Data points marked with hashmarks are significantly different from the corresponding treatment of the wild type cells (Bonferroni test; ## *p* < 0.01) (**A**). Cellular ATP was assayed 24 h after cisplatin treatment (**B**). Cells were treated with cisplatin alone or in combination with apoptosis (z-DEVD-FMK, 100 μM) or necroptosis (NEC1, 30 μM) inhibitors. Inhibitors were added 30 minutes before cisplatin treatment. Bars marked with asterisks are significantly different from the control (not shown) (Dunnett test; ** *p* < 0.01, *** *p* < 0.001). Bars marked with hashmarks are significantly different from the cisplatin-treated samples (Dunnett test; ## *p* < 0.01, ### *p* < 0.001). (**B**). Oxygen consumption rate (OCR)/oxidative phosphorylation (**C**) and extracellular acidification rate (ECAR)/glycolytic activity (**D**) were determined using a Seahorse metabolic analyzer and expressed as a percentage of the WT baseline. Both OCR/oxidative phosphorylation and ECAR/glycolytic activity were monitored in specific stress tests 13 h after cisplatin treatment. Mitochondrial stress tests included oligomycin (2 µM), FCCP (0.5 µM), and antimycin A (1 µM) for OCR (**C**) and glucose (10 mM), oligomycin (2 µM) and 2-deoxyglucose (50 mΜ) for ECAR (**D**). Data points marked with asterisks are significantly different from the corresponding vehicle treated data points at the same phase of the graph (Student–Newman–Keuls method; * *p* < 0.05). Data points marked with hashmarks are significantly different from the corresponding data points of wild type cells at the same phase of the graph (Student-Newman-Keuls method; # *p* < 0.05). Ratios of the two metabolic routes were determined from the baseline OCR and ECAR values 13 hours after cisplatin treatment. Bars marked with asterisks are significantly different from the corresponding vehicle treated group (Student-Newman-Keuls method; * *p* < 0.05). Bars marked with hashmarks are significantly different from the corresponding group of wild type cells (Student-Newman-Keuls method; # *p* < 0.05) (**E**). Data plotted are means ± SEM (*n* = 3).

Metabolic reprogramming is a hallmark of cancer. Moreover, anticancer agents, including cisplatin, have been reported to cause metabolic perturbations [33,34]. Considering that NAD^+^ is a central energy metabolite and NMNAT1 knockout resulted in a significant drop in cellular NAD^+^, we hypothesized that reduced NAD^+^ availability may have an impact on cell metabolism in cisplatin-treated U-2OS cells. Basal NAD^+^ levels were significantly lower in NMNAT1^−/−^ cells and slightly but significantly decreased by cisplatin treatment (Figure 4A). A more dramatic change in ATP levels was observed. Cisplatin caused a marked drop in cellular ATP content in NMNAT1 deficient cells while no change could be seen in the wild type cells (Figure 4B). Cell death-associated impairment of energy production was partly responsible for the drop in ATP level, as both the caspase inhibitor z-DEVD-FMK and the necroptosis inhibitor NEC1 significantly prevented ATP loss (Figure 4B).

To characterize cellular energy-producing pathways, we measured oxygen consumption rate, (OCR) as a measure of cellular respiration, and extracellular acidification rate (ECAR), as a measure of glycolysis. No major difference could be observed in basal respiration between the two cell lines (Figure 4C). A mitochondrial stress test, conducted by sequential addition of different mitochondrial toxins (see Materials and Methods section for details), also showed similar responses in both cell lines. However, cisplatin treatment completely reduced mitochondrial respiratory reserve capacity in NMNAT1^−/−^ cells and cells were unable to recover even basal respiratory activity after oligomycin treatment (Figure 4C). Wild type cells, on the other hand, displayed unaltered respiratory adaptation in response to cisplatin. The basal glycolytic activity proved to be significantly higher in the NMNAT1^−/−^ cell line (Figure 4D). However, no significant differences between the two cell lines could be observed in glycolytic stress tests with or without the addition of cisplatin. This finding suggests that glycolytic activity remains mostly unaffected by cisplatin regardless of the NMNAT1 status. Cellular metabolism can be characterized by the OCR/ECAR ratio, based on the basal rates of the two parameters. Knockout cells have a reduced OCR/ECAR ratio indicating that they are more glycolytic, compared to the WT cells (Figure 4E). Furthermore, cisplatin treatment caused a more pronounced drop in the basal OCR/ECAR ratios in the NMNAT1^−/−^ cells, demonstrating even more reliance on glycolysis rather than respiratory energy production.

### 2.4. The Role of PARylation in the Increased Cisplatin Sensitivity of NMNAT1^−/−^ K.O. Cells

**Figure 5:**
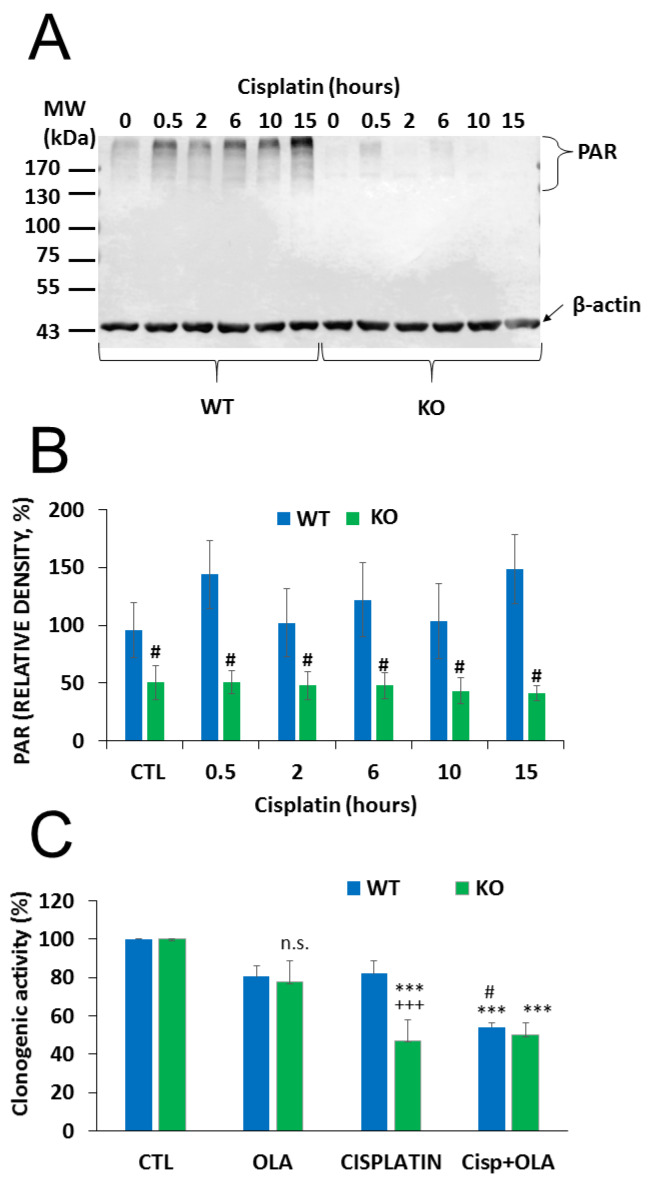
Impaired PARylation contributes to increased cisplatin sensitivity of NMNAT1^−/−^ cells. The level of poly(ADP-ribose) (PAR) polymer formation was detected using western blot in wild type (WT) and in NMNAT1 ^−/−^ (KO) cells at the indicated timepoints (0.5, 2, 6, 10, and 15 h) after cisplatin (5 μg/mL) treatment (**A**). Full WB image can be found in Supplementary Material. Actin was used as a loading control. Relative densities (normalized to beta actin and compared to the untreated WT control) are shown on panel (**B**). Bars marked with hashmarks are significantly different from the corresponding samples of wild type cells (Bonferroni test; # *p* < 0.05). The role of possible of PARP-1-NMNAT1 interaction in clonogenic activity was determined by counting crystal violet-stained colonies at day 6 (**C**). Cells were treated with vehicle (CTL), cisplatin, or the combination of cisplatin (5 μg/mL) and olaparib (10 μM) (Cisp+ OLA) and individual colonies were counted in each sample. Results are expressed as a percentage of the number of colonies in the vehicle treated samples. Bars marked with asterisks are significantly different from the corresponding control (Bonferroni test; *** *p* < 0.001). Bars marked with hashmarks are significantly different from the corresponding cisplatin treated samples (Bonferroni test; # *p* < 0.05). Bars marked with + are significantly different from the corresponding samples of wild type cells (Bonferroni test; +++ *p* < 0.001). Data plotted are means ± SEM (*n* = 3).

Cisplatin-induced DNA damage activates PARP1 [35] and PARP1 activation contributes to the repair of cisplatin-crosslinked DNA lesions and cell survival. Thus, we hypothesized that NMNAT1 deficiency and consequent nuclear NAD^+^ scarcity may compromise PARP1’s ability to initiate the DNA damage response and prevent cell death. We found that cisplatin caused a time dependent poly(ADP-ribose) (PAR) formation in WT cells, while no PAR formation could be detected in the NMNAT1^−/−^ cells (Figure 5A,B). Moreover, cisplatin treatment caused only a mild and non-significant decrease in the clonogenic activity of wild type cells, but significantly reduced the clonogenic activity of NMNAT1^−/−^ cells. Combined treatment with cisplatin and the PARP inhibitor (olaparib) caused a significantly lower proliferation in wild type cells, compared to the cisplatin-treated cells, but no further decrease could be detected in the NMNAT1^−/−^ cells (Figure 5C). These data suggest that cisplatin-induced DNA damage is unable to activate PARP1 in NMNAT1^−/−^ cells and substrate deprivation of PARP1 limits the capacity of the cells to cope efficiently with DNA injury.

### 2.5. Chemosensitization in 3D Cultures and in Combination Treatment

**Figure 6:**
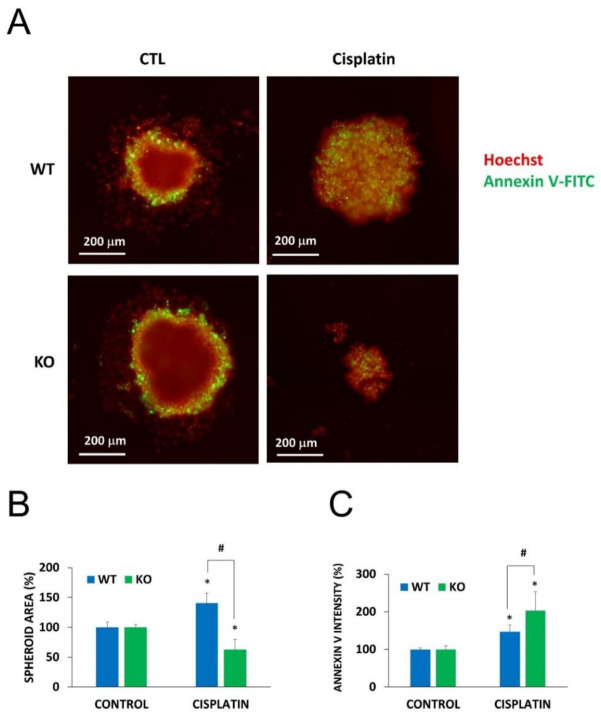
Chemosensitivity of spheroids to cisplatin treatment. Using WT and NMNAT-1^−/−^ U-2OS cells, 3D cell cultures (spheroids) were generated. Spheroids were treated with cisplatin (50 μg/mL) for 6 days. Cells were stained without fixation with Hoechst (red pseudocolor) and Annexin V (green). Representative images are shown in panel **A**. Images of 3 spheroids/treatment were taken and analyzed for area (**B**) and the intensity of the inner region (**C**). Results are expressed as percentages of the control (vehicle treated) samples. Bars marked with asterisks are significantly different from the vehicle treated control (Student-Newman-Keuls method; * *p* < 0.05). Bars marked with hashmarks are significantly different from the cisplatin treated wild type spheroids (Student-Newman-Keuls method; # *p* < 0.05). Data plotted are means ± SEM (*n* = 3).

To demonstrate the role of NMNAT1 in cellular models that more closely resemble in vivo conditions or clinical settings, we investigated a) chemosensitization to cisplatin by NMNAT1 deficiency in a 3D model (as opposed to 2D cultures) and b) a treatment protocol based on a combination of chemotherapeutic drugs. Wild type and NMNAT1^−/−^ U-2OS cells formed spheroids under favorable conditions (see “Materials and Methods” section for detail). Cell-to-cell contacts in spheroids render cells resistant to toxic stimuli [36]; therefore, we used higher concentrations of cisplatin in the spheroid experiments. While spheroids of the wild type cells only became less compact at 50 μg/mL cisplatin concentration, NMNAT1^−/−^ U-2OS cells displayed a significant decrease in size, or a complete disintegration of the spheroids under the same conditions (Figure 6A,B). The inner region of cisplatin-treated spheroids show a marked elevation in Annexin V positivity, a more dramatic elevation can be detected in the case of NMNAT1^−/−^ spheroids (Figure 6C).

**Figure 7:**
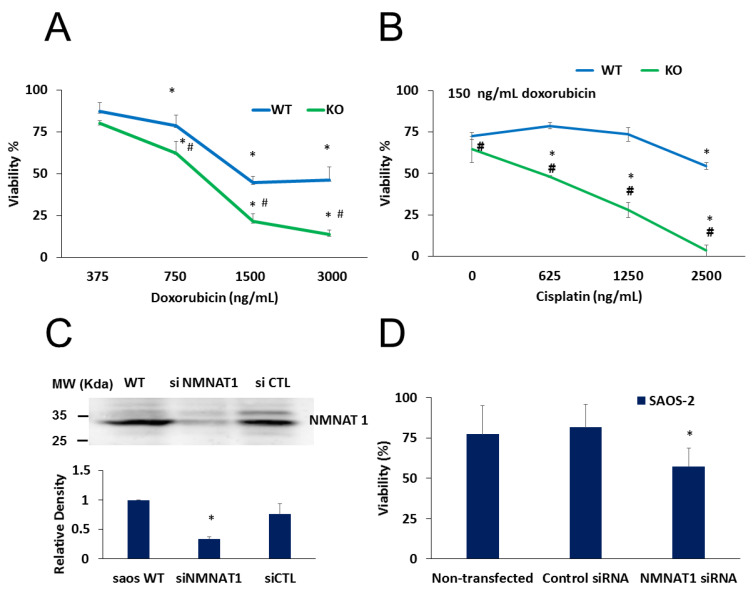
Combined treatments on U-2OS WT and NMNAT1^−/−^ cells, chemosensitivity of SAOS-2. Wild type (WT) and NMNAT1^−/−^ cells were treated with the indicated concentrations of doxorubicin (DOX) and viability was determined with the Calcein-AM assay 24 hours after DOX treatment. Data points marked with asterisks are significantly different from the corresponding vehicle treated data points (not shown) (Dunnett test; * *p* < 0.05). Data points marked with hashmarks are significantly different from the corresponding data points of wild type cells (Student–Newman–Keuls method; # *p* < 0.05) (**A**). DOX was also applied in combination with cisplatin (**B**). Panel B shows the viability of WT and KO at constant (150 ng/mL) DOX and increasing cisplatin concentrations. Data points marked with asterisks are significantly different from the corresponding vehicle treated data points (Dunnett test; * *p* < 0.05). Data points marked with hashmarks are significantly different from the corresponding data points of wild type cells (Student–Newman–Keuls method; # *p* < 0.05). SAOS-2 cells were transfected with control or NMNAT-1 specific siRNAs. Efficiency of NMNAT-1 silencing was verified by Western blot (**C**). Full WB image can be found in Supplementary Material. Cells were treated with cisplatin for 48 hours and a Calcein-AM viability assay was performed (**D**). Data points marked with asterisks are significantly different from the vehicle-treated sample (Dunnett test; * *p* < 0.05). Data plotted are means ± SEM (*n* = 3).

Moreover, a combination of cisplatin with doxorubicin resulted in more efficient killing of cancer cells (Figure 7). A concentration series of doxorubicin was used to explore differences in the sensitivity of wild type and NMNAT1^−/−^ U-2OS cells. At concentrations higher than 750 ng/mL, knockout cells were significantly more sensitive to doxorubicin (Figure 7A). Cells were exposed to different concentrations of cisplatin and a fixed doxorubicin concentration of 150 ng/ml. Combination treatment was significantly more efficient in killing NMNAT1^−/−^ cells than wild type cells (Figure 7B).

To confirm the sensitizing effect of the absence of NMNAT1 in cisplatin-induced cell death, cells of another osteosarcoma cell line, SAOS-2, were used. NMNAT1 could be effectively silenced in SAOS-2 cells (Figure 7C). Similarly to NMNAT-1 knockout U-2OS cells, NMNAT-1 silenced SAOS-2 cells also showed significantly higher sensitivity to cisplatin treatment (Figure 7D).

## 3. Discussion

Similar to most neoplastic diseases, osteosarcoma poses a great challenge to modern medicine. The therapeutic approaches are limited and are based on the combination of chemotherapy and surgical removal of the primary tumor and metastasis. Two of the most efficient chemotherapeutics used for the therapy of osteosarcoma (cisplatin and doxorubicin) cause DNA injury. Methotrexate, the third component of the most common chemococktails, is an anti-metabolite folate analog that interferes with the synthesis of purine and pyrimidine bases and, therefore, DNA and RNA synthesis. Of note, the anticancer effect of methotrexate has also been reported to be due to DNA breakage [37]. Thus, it seems that the DNA damage response (DDR) might be the Achilles heel of osteosarcomas. Targeting the DNA damage response is a novel anticancer approach in cancers displaying DNA repair defects. The best known example is PARPi treatment of BRCA-negative ovarian cancers [38]. Nuclear NAD^+^ synthesis may be an unlikely target of DNA repair in cancer therapy. However, as the substrate of PARP enzymes, NAD^+^ may be a limiting factor for PARylation activity. Moreover, the nuclear SIRT enzymes, another major NAD^+^ consumer group, are also known to promote cell survival and are potential cancer targets (see more details below).

Previous experiments with NAMPT inhibitors suggested that inhibition of the salvage pathway of NAD^+^ synthesis may have beneficial effects for cancer therapy [39]. Due to the lack of specific NMNAT1 inhibitors, however, the role of NMNAT1 in cancer has not yet been investigated. In the present study, we have investigated the role of NMNAT1 as a potential target in osteosarcoma. All cancer cell lines that were tested expressed NMNAT1 mRNA. Compared to the other assayed cell lines, U-2OS osteosarcoma cells displayed an average level of NMNAT1 mRNA. Our mRNA data also show that in U-2OS cells, the NMNAT-1 has the highest expression, while NMNAT2 and NMNAT-3 have lower contributions. These cells tolerated inactivation of the NMNAT1 gene without showing decreased viability or marked morphological alterations. Notable phenotypic differences between NMNAT1^−/−^ and wild type cells included reduced proliferation capacity (clonogenicity) and moderately increased glycolytic activity. The most prominent feature of the NMNAT1^−/−^ cells was a markedly reduced NAD^+^ level, which did not compromise cellular energy production in the resting state, as reflected by the unaltered ATP content. The two-thirds reduction in cellular NAD^+^ in NMNAT1^−/−^ cells highlights the dominant role of NMNAT1 in cellular NAD^+^ synthesis, as elevated NMNAT-2 expression cannot substitute the role of NMNAT1 in NAD+ production of NMNAT1 KO cells. A cross-compartment exchange of NAD^+^ between nuclear and cytoplasmic pools is possible, as demonstrated in a model of adipogenic signaling where NMNAT2 activation drained nuclear NAD^+^ while competing for the common substrate NMN [40]. Similar nuclear and cytoplasmic NAD^+^ concentrations also suggest relatively barrier-free equilibration of NAD^+^ between these compartments [41]. However, deciphering mechanisms underlying the marked drop of total NAD^+^ in NMNAT1^−/−^ cells and characterization of the effect of NMNAT1 inactivation on the main NAD^+^ compartments require further investigation.

A key question asked in our study was whether NMNAT1 contributes to the cells’ ability to cope with DNA damage caused by chemotherapeutic agents used in osteosarcoma therapy. Induction of NMNAT1 by cisplatin and doxorubicin suggests that NAD^+^-dependent processes may be involved in the cellular survival response to these chemotherapeutics. Moreover, a major finding of our paper is that NMNAT1 targeting is a potent chemosensitizing strategy. NMNAT1^−/−^ U-2OS cells displayed increased sensitivity to cisplatin, as indicated by reduced viability and increased cell death compared to wild type U-2OS cells. Cisplatin-induced cell death of NMNAT1 knockout cells showed features of both apoptosis and necroptosis. Activation of caspase3/7-like proteases and the cytoprotective effect of caspase-3/7 inhibition suggest that caspase-mediated apoptosis is involved in cell death in our model. Furthermore, the protective effect of the necroptosis inhibitor Nec1 indicates that the necroptotic pathway is also activated and contributes to cell death. If cisplatin-induced killing of NMNAT1 deficient cells also has a necroptosis-like component in vivo, NMNAT1 inhibition may enhance the anticancer immune response or may promote metastasis [42]. A detailed characterization of cell death modalities involved in the killing of osteosarcoma cells with reduced nuclear NAD^+^ production and the effects on antitumor immunity versus metastasis formation goes beyond the scope of the current paper.

An important question raised by our study is whether the anticancer potential of NMNAT1 targeting is restricted to osteosarcoma. It is quite likely that targeting NMNAT1 may also be beneficial in other types of tumors. In support of this statement, we found that NMNAT1 is likely to be important for the progression of various tumors. RNAsec data from the kmplot database [43] show that low NMNAT1 expression correlates with better survival of patients with sarcomas, liver hepatocellular carcinoma, bladder carcinoma, breast cancer, esophageal adenocarcinoma, kidney renal papillary cell carcinoma, pancreatic ductal adenocarcinoma and uterine corpus endometrial carcinoma (Figure S3). Whether NMNAT1 inhibition provides therapeutic benefits in these and other tumors requires further investigation.

Our data strongly support the role of PARP1 in the chemosensitization process. We hypothesize that nuclear NAD^+^ levels may be too low to support PARP activity in NMNAT1^−/−^ cells. In fact, cisplatin stimulated PARylation in wild type cells. However, PAR formation was absent in NMNAT1^−/−^ cells. Moreover, the potent PARP inhibitor olaparib enhanced the anti-clonogenic effect of cisplatin in the wild type but not the NMNAT1-deficient cells. The role of PARP-1 in the anti-clonogenic effect is also supported by our unpublished data from experiments with PARP-1 and PARP-2 silenced osteosarcoma cells. These results suggest that PARP1 activation acts as a survival factor via assisting DNA repair, in line with our current understanding of the role of PARylation in the repair of cisplatin-induced DNA damage [44]. PARP1 is unable to contribute to cell survival in NMNAT1^−/−^ cells (due to the shortage of NAD^+^). Thus, a key factor in the chemosensitizing effect of NMNAT1 inactivation is reduced PARylation and, consequently, impaired DNA repair. It is tempting to hypothesize that NMNAT1 and PARP1 interact directly at sites of DNA damage, similar to the direct interaction between NMNAT1 and PARP1 on PARP1-dependent promoters [45]. This interaction might involve the binding of NMNAT1 to PAR polymers [46]. This hypothesis, however, needs experimental verification. The possible role of PARP1 in the chemosensitization of NMNAT1^−/−^ osteosarcoma cells was also supported by previous preclinical studies. These papers reported (a) correlation between PARP1 expression level and osteosarcoma cell survival; (b) osteosarcoma cell killing or chemosensitization by PARP1 knockout or PARP inhibition [47,48,49]. Ongoing and finished clinical trials (e.g., ClinicalTrials.gov ID: NCT01583543, NCT01858168 NCT02044120) also set out to investigate the effects of olaparib and other PARP inhibitors in osteosarcoma. Our data suggest that, at least under our current experimental settings, much of the chemosensitization observed in NMNAT1 cells is due to indirect inhibition of PARylation. It remains to be seen, however, how potent NMNAT1 inhibitors (which are not yet available) will compare with clinically used PARPi compounds in terms of therapeutic spectrum, efficiency, tolerability and side effects in different types of tumors. We hypothesize that PARPi and NMNAT1i will necessarily differ in one or more of these aspects.

The hypothesis that impaired metabolic adaptation may also play a role in the chemosensitivity of NMNAT1-deficient cells is plausible, considering the central role of NAD^+^ in energy metabolism. Under basal conditions, a slight shift from respiration towards glycolysis could be detected in NMNAT1^−/−^ cells but energy production was balanced, as indicated by the normal ATP levels. DNA damage revealed the vulnerability of the respiratory system, as the mitochondrial stress test identified severely impaired respiratory reserve capacity. Multiple lines of evidence highlight the importance of metabolic regulation in tumor behavior and chemosensitivity. For example, analysis of integrated transcriptomic and metabolomics datasets identified altered glycolysis-related mRNAs and metabolites in osteosarcoma samples [50]. Moreover, various microRNAs regulate both osteosarcoma growth (proliferation) and glycolysis either positively (e.g., miRNA543) [51] or negatively (e.g., miRNA186) [52]. Furthermore, glycolysis also appears to correlate with chemosensitivity (e.g., cisplatin sensitivity [53]) or viability of osteosarcoma cells [54,55].

The extent to which SIRT inhibition contributes to chemosensitization under NAD^+^ scarcity is worth investigating. The nucleus is home to several sirtuin (SIRT) enzymes (SIRT1,6,7). SIRT1 plays a role in the DNA damage response [56]. Furthermore, the central role of sirtuins in energy homeostasis [57] and metabolic regulation [58] support the possible involvement of sirtuins in shifting metabolic balance (e.g., respiration vs glycolysis) after DNA damage. Nuclear SIRTs facilitate cell survival under various stress conditions, underlining their potential role in chemosensitization. For example, SIRT6 is downregulated in DOX-treated liver cancer cells, contributing to DOX-induced cell death. The pro-survival role of SIRT6 in this model, as demonstrated by the overexpression of the enzyme, is mediated by Foxo3 degradation [59]. Inhibition of SIRT6 also sensitizes cancer cells to chemotherapy [60]. Similarly, SIRT7 has been identified as a survival factor via complex mechanisms including direct p53 diacetylation [61] and the promotion of autophagy [62]. In a transcription regulatory setting, a requirement for NMNAT1 and NAMPT for SIRT1 activity has already been demonstrated [63] and similar interplay between nuclear SIRTs and enzymes of the salvage pathway of NAD^+^ synthesis is likely to take place in the DNA damage response as well. Nevertheless, clarifying the role of SIRTs in the chemosensitizing effect of NMNAT1 inactivation goes beyond the scope of this paper.

In summary, NMNAT1 has been identified as a possible target for the treatment of osteosarcoma. DNA damaging chemotherapeutic agents are likely to cause synergistic toxicity with NMNAT1 inhibitors, if NMNAT1 inhibitors are developed. Key mechanisms underlying this synergy include the impairment of PARylation-dependent DNA repair processes and metabolic adaptation. The chemosensitization by NMNAT1 inactivation could also be observed under more clinically relevant conditions, i.e., in 3D spheroids and combination chemotherapy. However, the effect of NMNAT1 targeting in non-transformed cells is a critical issue for the development of anticancer strategies targeting NMNAT1.

## 4. Materials and Methods

### 4.1. D Cell Culture

Human U-2 OS cells were grown in Dulbecco’s modified Eagle’s medium (DMEM, #12-604F, Lonza, Basel, Switzerland), containing 10% fetal bovine serum (#10500-064, GIBCO, ThermoFisher Waltham, MA, USA) L-glutamine, and penicillin-streptomycin, under standard cell culture conditions (humidified atmosphere, 5% CO_2_). The cells were routinely tested for mycoplasma contamination.

### 4.2. D Cell Culture (Spheroids)

Spheroids were grown as previously described [64] with modifications as follows. Flat-bottom cell culture microplates were coated with low melting point agarose to form a U-shaped, cell-repellent bottom. Cells were seeded into the wells and grown for 2 days to form spheroids.

### 4.3. Generation of NMNAT1^−/−^ Cells by Crispr-cas9

NMNAT1 knock-out U-2OS cells were made by CRISPR-Cas9 technology using reagents from Santa Cruz Biotechnology, following the manufacturer’s instructions. Cells were grown to 40–80% confluence and then trypsinized in antibiotic-free standard growth medium (DMEM, #12-604F, Lonza, Basel, Switzerland). Cells were counted, cell number was adjusted to 5.33 × 10^5^ cells/mL, and a 2 mL cell suspension was centrifuged (100× *g*, 10 min). UltraCruz^®^ Transfection Reagent (18 µL; sc-395739, Santa Cruz Biotechnology, Dallas, TX, USA) was diluted with 82 µL of plasmid transfection medium (sc-108062, Santa Cruz Biotechnology, Dallas, TX, USA) to bring the final volume to 100 µL. The medium was carefully aspirated and the diluted transfection reagent was pipetted onto the cells. Next, 2.5 µL of NMNAT1 CRISPR Plasmid and 2.5 µL of HDR Plasmid were added to the tube. Cells were immediately transferred to transfection cuvettes and transfected using an Amaxa Nucleofector II (Lonza, Basel, Switzerland). Transfected cells were pipetted into 6-well plates, which contained pre-warmed cell culture media (DMEM, #12-604F, Lonza, Basel, Switzerland) supplemented with 10% fetal bovine serum (#10500-064, GIBCO, ThermoFisher Waltham, MA, USA), 5% L-glutamine, and 5% penicillin-streptomycin. Cells were incubated for 24–72 hours under regular conditions and transfection efficiency was visually confirmed by the detection of red fluorescent protein (RFP) via fluorescent microscopy. Transfected cells were selected for puromycin (2.5 μg/mL) resistance for 3 weeks and sorted for RFP fluorescence with a BD LSR II Cell Sorter (Franklin Lakes, NJ, USA. Single-cell colonies were grown and used for the experiments. The NMNAT1 expression of the clones was checked with qPCR for mRNA-level and Western blot for protein level (see below).

### 4.4. Western Blot

Western blotting was carried out as previously described [65,66,67], with modifications as follows. U-2OS cells were sonicated in RIPA lysis buffer supplemented with protease inhibitor cocktail (#M221, VWR International, Radnor, PA, USA) and phosphatase inhibitor (PMSF, 1:100; # PMSF-RO, Merck, Darmstadt, Germany). Lysates were centrifuged at 16,100× *g* for 10 min at 4 °C. Protein concentrations of the supernatants were measured with a Direct Detect infrared spectrometer (EMD Millipore Corporation, Burlington, MA, USA). Equal amounts of the proteins from each extract were separated on SDS-polyacrylamide gels (10%) in SDS-Tris-glycine running buffer (10× glycine, Tris, SDS). The proteins were then transferred electrophoretically to nitrocellulose membranes in transfer buffer (5× Tris, glycine, in dH_2_O). Membranes were blocked for 1 hour at room temperature with 5% skim milk-powder (#70166, Merck, Darmstadt, Germany) dissolved in 0.01% PBS-Tween20. Primary antibodies were applied in blocking solution overnight at 4 °C. Horseradish peroxidase (HRP)-coupled secondary antibodies were applied in the same type of solution as primary antibodies for 2 hours at room temperature. ECL-based chemiluminescence (Super Signal, West Pico Plus, Luminol/Enhancer #1863098, Peroxidase Solution #1863099, ThermoFisher Waltham, MA, USA) was used for detection. Images were acquired with a Bio-Rad ChemiDoc Imager (Bio-RAD, Hercules, CA, USA) and ImageLab 6.0 software was used for protein quantification. The antibodies used for western blotting are shown in Table S1.

### 4.5. High Content Analysis (HCA)

#### 4.5.1. HCA on Fixed Cells: Quantification of γH2AX

Cells (2 × 10^4^, 100 μL/well) were seeded into sterile microplates (Cell Carrier-96 ultra, PerkinElmer, Waltham, MA, USA) and grown for 24 h. After applying the indicated cell treatments (see figure legends), cells were fixed in 3% formaldehyde/PBS solution for 15 min at room temperature, washed 3× with PBS, and incubated in blocking solution (5% BSA in PBS) for 15 min at room temperature. The anti-phospho-H2AX antibody (Table S2) was diluted in blocking solution and added to wells (50 μL/well, 2 h at room temperature). The secondary antibody (Alexa Fluor 488, Table S2) was diluted in blocking solution and incubated for 1 hour. After antibody incubations, cells were washed twice with PBS and incubated for 5 min with PBS containing 4’,6-diamidino-2-phenylindole dihydrochloride (DAPI, Table S2) at room temperature for nuclear staining. Cells were then washed three times with PBS and were kept in 100 μL of PBS until imaging. Images were acquired using an Opera Phenix High Content Analyzer (PerkinElmer, Waltham, MA, USA) with a 10× air objective (NA 0.3). Image analysis was performed with the built in Harmony software (version 4.8).

#### 4.5.2. HCA on Live Cells: Caspase Activation

Cells (2 × 10^4^ cells, 100 μL/well) were seeded into 96-well plates (Perkin Elmer, Waltham, MA, USA) and grown for 24 h. The following day, cells were pretreated with 100 μM z-DEVD-FMK caspase inhibitor (#S7312, Selleckchem, Houston, TX, USA) and/or treated with 5 µM cisplatin (Accord, Warsaw, Poland). Then, the cells were stained with 50 μL CellEvent™ Caspase-3/7 Green Detection Reagent (Table S2) in 7 μM final concentration. The plates were placed into an Opera Phenix High-Content Analyzer (Perkin Elmer, Waltham, MA, USA) with environment control (5% CO_2_, 37 °C) and fluorescence was measured every hour for 11 h at 502/530 nm. The analysis was performed with the Harmony software (Perkin Elmer, Waltham, MA, USA).

#### 4.5.3. HCA on Live Cells: Cell Death in Spheroids

Spheroids were transferred to glass bottom microplates (Cell Carrier-96 ultra, PerkinElmer, Waltham, MA, USA) and treated with cisplatin (25, 50, and 100 μg/mL) on day 2 and day 4. Afterward, the cells were stained with Hoechst (Table S2) and Annexin V-FITC (Table S2.) for 1 hour in growth medium. Images were acquired using an Opera Phenix High Content Analyzer (Perkin Elmer, Waltham, MA, USA). Fluorescence intensity was detected at 350 (Hoechst) and 488 nm (Annexin V-FITC).

#### 4.5.4. HCA on Live Cells: Cell Morphology

Cells (2 × 10^4^ cells, 100 μL/well) were seeded into 96-well plates (Perkin Elmer, Waltham, MA, USA) and grown for 24 h. The following day, cells were stained with 50 μL DRAQ5 (Table S2.) to obtain a final concentration of 2.5 μM. Plates were placed into an Opera Phenix High-Content Analyzer with environment control (5% CO_2_, 37 °C), fluorescence was measured at 488 to 647 nm, and the analysis was performed with the Harmony software (version 4.8).

### 4.6. Calcein-AM Viability Assay

This assay was carried out as previously described [68], with modifications as follows. Cells (2 × 10^4^ cells, 100 μL/well) were seeded into 96-well plates and were grown for 24 h. The next day, cells were treated with the indicated treatments (see figure legends). Two different batches of cisplatin were used for the experiments. The first batch was used for the experiments presented on Figure 1 (Figure 1B–D), whereas a second batch was used for the experiments presented in Figure 3, Figure 4, Figure 5, Figure 6, and Figure 7. Then the cells were stained by adding 50 μL of Calcein-AM (#17783, Merck, Darmstadt, Germany) solution in a final concentration of 1 μM. After incubating cells for 1 h at 37 °C, the fluorescent signal was measured with a Tecan Spark 20M (Tecan, Männedorf, Switzerland) multimode reader (Ex/Em = 485/530 nm with). Viability was expressed as a percentage of the untreated control.

### 4.7. Clonogenic Survival Assay

U-2OS cells were seeded into 6-well plates (#92006, TPP, Trasadingen, Switzerland) at a density of 1 × 10^3^ cells/mL. After 24 h the cells were treated with 10 μM PJ34 (P4365, Merck, Darmstadt, Germany) or 10 μM Olaparib (S1060, Selleckchem, Houston, TX, USA) for 30 minutes, and then with 5 μg/mL cisplatin (Accord Healthcare Inc., Durham, UK). Cells were incubated for 6 days and then counted manually after staining with 0.5% crystal violet dissolved in 20% ethanol [69].

### 4.8. Lactate Dehydrogenase (LDH) Release Assay

Cell death was assessed by determining the activity of LDH released into the culture medium. The LDH assay kit (#786-210, GBiosiences, St. Louis, MO, USA) was used as described by the manufacturer.

### 4.9. Measurement of Total Cellular NAD^+^ and ATP

NAD^+^ content was measured with an NAD^+^ assay kit following the manufacturer’s instructions (NAD^+^/NADH Quantitation Kit. #MAK037-1KT, Merck, Darmstadt, Germany). The NAD^+^ content of the cells was measured with a plate reader (Tecan Spark 20M, Tecan, Männedorf, Switzerland) at 450 nm.

ATP content was determined using an ATP assay kit following the manufacturer’s instructions (#110M6101, Merck, Darmstadt, Germany). Luminescence was measured with a plate reader (Tecan Spark 20M, Tecan, Männedorf, Switzerland). NAD^+^ content and ATP content were normalized to protein content.

### 4.10. Metabolic Analysis with Seahorse Metabolic Analyzer

U-2OS cells were seeded (1 × 10^5^ cells/well) in DMEM in Seahorse XF96 cell culture microplates (Agilent Technologies, Inc., Santa Clara, CA, USA) and incubated overnight at 5% CO_2_ and 37 °C. The sensor cartridge was prepared by adding 200 µL of dH_2_O overnight. Then, Seahorse Bioscience XF96 calibrant solution (pH 7.4) (Part No.: 100-840-000) was added to each well of a Seahorse 96-well utility plate. The sensors with the calibrant solution were incubated for 2 hours at 37 °C without CO_2_. The measurement was performed using the Seahorse XF96 Analyzer (Agilent Technologies, Inc., Santa Clara, CA, USA).

XF Cell Mito Stress analyses were performed as described in [70], with modifications as follows. The mitochondrial inhibitors were applied at the following final concentrations: 2 µM oligomycin, 0.5 µM FCCP, and 1 µM antimycin-A. Glycolysis stress was based on [70].

### 4.11. Sulforhodamine B (SRB) Assay

The SRB assay was performed as described in Kovács, P. et al. [71].

### 4.12. RNA Extraction and Quantitative PCR

RNA was purified with TRIzol reagent (Tri-RNA reagent, #FATRR001, Amplicon, Odense, Denmark) as described in the manufacturer’s instructions. Quantitative real-time PCR was performed with a LightCycler 480 thermocycler (Roche, Basel, Switzerland) using SYBR Green (SyberGreen, #4472908, Applied Biosystems, Foster City, CA, USA) according to the manufacturer’s protocol. Reactions were carried out in triplicate and data were normalized to the geometric mean of housekeeping genes (36B4 and cyclophilin). Sequences of primers are given in Table S3. hNMNAT1, hNMNAT2, hNMNAT3, hATP5B, hHSP60, hPKM2 primers were ordered from IDT (Coralville, IA, USA); and h36B4 and hcyclophilin from Merck (St. Louis, MO, USA).

### 4.13. Statistical Analysis

Experiments were repeated at least three times and data are expressed as mean ± SEM. Based on the type of experiment and the distribution of data, different kinds of statistical analysis were used. The statistical tests used are indicated in the figure legends.

## 5. Conclusions

Genetic inactivation of NMNAT1 sensitizes U-2OS osteosarcoma cells to cisplatin, doxorubicin, or a combination of these two treatments. Upon cisplatin treatment, an impaired PARP1 activity, insufficient DNA repair, a marked drop in cellular ATP and a limited mitochondrial reserve capacity could be observed, which all contribute to the higher chemosensitivity of NMNAT-1^−/−^ cells. Increased cell death of NMNAT1^−/−^ cells shows features of both apoptosis and necroptosis. NMNAT1^−/−^ cells also displayed markedly higher sensitivity to cisplatin when grown as spheroids in 3D culture. Our results suggest that NMNAT1 may be worth investigating further as a potential target in cancer therapy.

## Supplementary Materials

The following are available online at https://www.mdpi.com/2072-6694/12/5/1180/s1, Figure S1: mRNA expressions of NMNAT enzymes and induction of NMNAT1 protein expression in U-2OS cells, Figure S2: Cell morphology analysis, Figure S3: Tumors with better patient survival at low NMNAT1 expression, Table S1: Antibodies used for Western blotting, Table S2: Antibodies and dyes used for High Content Analysis and Table S3: Sequences of primers, used in quantitative PCR experiments. Supplementary materials also contain the full images of Western Blots.

## References

[1] Steliarova-Foucher E., Colombet M., Ries L.A.G., Moreno F., Dolya A., Bray F., Hesseling P., Shin H.Y., A Stiller C., Bouzbid S. (2017). International incidence of childhood cancer, 2001–10: A population-based registry study. Lancet Oncol..

[2] Mendoza P.R., Grossniklaus H.E. (2015). The Biology of Retinoblastoma. Prog. Mol. Biol. Transl. Sci..

[3] Santibáñez-Koref M., Birch J., Hartley A., Crowther D., Harris M., Jones P.M., Kelsey A., Craft A., Eden T. (1991). p53 germline mutations in Li-Fraumeni syndrome. Lancet.

[4] Lu L., Jin W., Liu H., Wang L.L. (2014). RECQ DNA Helicases and Osteosarcoma. Adv. Exp. Med. Biol..

[5] Buondonno I., Gazzano E., Tavanti E., Chegaev K., Kopecka J., Fanelli M., Rolando B., Fruttero R., Gasco A., Hattinger C.M. (2018). Endoplasmic reticulum-targeting doxorubicin: A new tool effective against doxorubicin-resistant osteosarcoma. Cell. Mol. Life Sci..

[6] Jimmy R., Stern C., Lisy K., White S. (2017). Effectiveness of mifamurtide in addition to standard chemotherapy for high-grade osteosarcoma. JBI Database Syst. Rev. Implement. Rep..

[7] (2020). Osteosarcoma—Childhood and Adolescence: Statistics. https://www.cancer.net/cancer-types/osteosarcoma-childhood-and-adolescence/statistics.

[8] Pavlova N., Thompson C.B. (2016). The Emerging Hallmarks of Cancer Metabolism. Cell Metab..

[9] Kim M.Y., Zhang T., Kraus W.L. (2005). Poly(ADP-ribosyl)ation by PARP-1: ‘PAR-laying’ NAD+ into a nuclear signal. Genes Dev..

[10] Lee H.C., Aarhus R. (1995). A Derivative of NADP Mobilizes Calcium Stores Insensitive to Inositol Trisphosphate and Cyclic ADP-ribose. J. Boil. Chem..

[11] Chini E., Dousa T. (1995). Enzymatic-Synthesis and Degradation of Nicotinate Adenine Dinucleotide Phosphate (NAADP), a Ca2+-Releasing Agonist, in Rat Tissues. Biochem. Biophys. Res. Commun..

[12] Cantó C., Sauve A.A., Bai P. (2013). Crosstalk between poly(ADP-ribose) polymerase and sirtuin enzymes. Mol. Asp. Med..

[13] Eisemann T., Pascal J.M. (2019). Poly(ADP-ribose) polymerase enzymes and the maintenance of genome integrity. Cell. Mol. Life Sci..

[14] Virág L. (2013). 50Years of poly(ADP-ribosyl)ation. Mol. Asp. Med..

[15] Hegedűs C., Virág L. (2014). Inputs and outputs of poly(ADP-ribosyl)ation: Relevance to oxidative stress. Redox Boil..

[16] Hottiger M.O., O Hassa P., Lüscher B., Schüler H., Koch-Nolte F. (2010). Toward a unified nomenclature for mammalian ADP-ribosyltransferases. Trends Biochem. Sci..

[17] Wang L., Liang C., Li F., Guan D., Wu X., Fu X., Lu A., Zhang G. (2017). PARP1 in Carcinomas and PARP1 Inhibitors as Antineoplastic Drugs. Int. J. Mol. Sci..

[18] Bürkle A., Virág L. (2013). Poly(ADP-ribose): PARadigms and PARadoxes. Mol. Asp. Med..

[19] Bryant H.E., Schultz N., Thomas H.D., Parker K.M., Flower D., Lopez E., Kyle S., Meuth M., Curtin N.J., Helleday T. (2005). Specific killing of BRCA2-deficient tumours with inhibitors of poly(ADP-ribose) polymerase. Nature.

[20] Farmer H., McCabe N., Lord C.J., Tutt A.N.J., Johnson D.A., Richardson T.B., Santarosa M., Dillon K.J., Hickson I., Knights C. (2005). Targeting the DNA repair defect in BRCA mutant cells as a therapeutic strategy. Nature.

[21] Calabrese C.R., Almassy R., Barton S., Batey M.A., Calvert A.H., Canan-Koch S., Durkacz B.W., Hostomsky Z., Kumpf R.A., Kyle S. (2004). Anticancer chemosensitization and radiosensitization by the novel poly(ADP-ribose) polymerase-1 inhibitor AG14361. J. Natl. Cancer Inst..

[22] Plummer R., Lorigan P.C., Steven N., Scott L., Middleton M.R., Wilson R.H., Mulligan E., Curtin N.J., Wang D., Dewji R. (2013). A phase II study of the potent PARP inhibitor, Rucaparib (PF-01367338, AG014699), with temozolomide in patients with metastatic melanoma demonstrating evidence of chemopotentiation. Cancer Chemother. Pharmacol..

[23] Curtin N.J., Szabo C. (2013). Therapeutic applications of PARP inhibitors: Anticancer therapy and beyond. Mol. Asp. Med..

[24] Gossmann T.I., Ziegler M. (2014). Sequence divergence and diversity suggests ongoing functional diversification of vertebrate NAD metabolism. DNA Repair.

[25] Chiarugi A., Dölle C., Felici R., Ziegler M. (2012). The NAD metabolome — a key determinant of cancer cell biology. Nat. Rev. Cancer.

[26] Bajrami I., Kigozi A., Van Weverwijk A., Brough R., Frankum J., Lord C.J., Ashworth A. (2012). Synthetic lethality of PARP and NAMPT inhibition in triple-negative breast cancer cells. EMBO Mol. Med..

[27] Berger F., Lau C., Dahlmann M., Ziegler M. (2005). Subcellular Compartmentation and Differential Catalytic Properties of the Three Human Nicotinamide Mononucleotide Adenylyltransferase Isoforms. J. Boil. Chem..

[28] Yamamoto M., Hikosaka K., Mahmood A., Tobe K., Shojaku H., Inohara H., Nakagawa T. (2016). Nmnat3 Is Dispensable in Mitochondrial NAD Level Maintenance In Vivo. PLoS ONE.

[29] Michishita E., Park J.Y., Burneskis J.M., Barrett J.C., Horikawa I. (2005). Evolutionarily Conserved and Nonconserved Cellular Localizations and Functions of Human SIRT Proteins. Mol. Boil. Cell.

[30] Barraud M., Garnier J., Loncle C., Gayet O., Lequeue C., Vasseur S., Bian B., Duconseil P., Gilabert M., Bigonnet M. (2016). A pancreatic ductal adenocarcinoma subpopulation is sensitive to FK866, an inhibitor of NAMPT. Oncotarget.

[31] Haubrich B.A., Ramesha C., Swinney D.C. (2019). Development of a Bioluminescent High-Throughput Screening Assay for Nicotinamide Mononucleotide Adenylyltransferase (NMNAT). SLAS Discov. Adv. Life Sci. R & D.

[32] Huang X., Okafuji M., Traganos F., Luther E., Holden E., Darzynkiewicz Z. (2004). Assessment of histone H2AX phosphorylation induced by DNA topoisomerase I and II inhibitors topotecan and mitoxantrone and by the DNA cross-linking agent cisplatin. Cytom. Part A J. Int. Soc. Anal. Cytol..

[33] Spincemaille P., Alborzinia H., Dekervel J., Windmolders P., Van Pelt J., Cassiman D., Cheneval O., Craik D.J., Schur J., Ott I. (2014). The Plant Decapeptide OSIP108 Can Alleviate Mitochondrial Dysfunction Induced by Cisplatin in Human Cells. Molecules.

[34] Rytelewski M., Tong J.G., Buensuceso A., Leong H., Vareki S.M., Figueredo R., Di Cresce C., Wu S.Y., Herbrich S., Baggerly K.A. (2014). BRCA2 inhibition enhances cisplatin-mediated alterations in tumor cell proliferation, metabolism, and metastasis. Mol. Oncol..

[35] Gunn A.R., Banos-Pinero B., Paschke P., Sanchez-Pulido L., Ariza A., Day J., Emrich M., Leys D., Ponting C.P., Ahel I. (2016). The role of ADP-ribosylation in regulating DNA interstrand crosslink repair. J. Cell Sci..

[36] An S.S.A., Seo O.W., Lee J., Hulme J., Baek N. (2016). Real-time monitoring of cisplatin cytotoxicity on three-dimensional spheroid tumor cells. Drug Des. Dev. Ther..

[37] Xie L., Zhao T., Cai J., Su Y., Wang Z., Dong W. (2016). Methotrexate induces DNA damage and inhibits homologous recombination repair in choriocarcinoma cells. Onco Targets Ther..

[38] López-Camarillo C., Rincón D.G., Ruiz-García E., De La Vega H.A., Marchat L.A. (2019). DNA Repair Proteins as Therapeutic Targets in Ovarian Cancer. Curr. Protein Pept. Sci..

[39] Cole J., Guiot M.-C., Gravel M., Bernier C., Shore G.C., Roulston A. (2017). Novel NAPRT specific antibody identifies small cell lung cancer and neuronal cancers as promising clinical indications for a NAMPT inhibitor/niacin co-administration strategy. Oncotarget.

[40] Ryu K.W., Nandu T., Kim J., Challa S., DeBerardinis R.J., Kraus W.L. (2018). Metabolic regulation of transcription through compartmentalized NAD+biosynthesis. Science.

[41] Cambronne X.A., Stewart M.L., Kim N., Jones-Brunette A.M., Morgan R.K., Farrens D.L., Cohen M.S., Goodman R.H. (2016). Biosensor reveals multiple sources for mitochondrial NAD+. Science.

[42] Gong Y., Fan Z., Luo G., Yang C., Huang Q., Fan K., Cheng H., Jin K., Ni Q., Yu X.-J. (2019). The role of necroptosis in cancer biology and therapy. Mol. Cancer.

[43] Nagy A., Lánczky A., Menyhart O., Győrffy B. (2018). Validation of miRNA prognostic power in hepatocellular carcinoma using expression data of independent datasets. Sci. Rep..

[44] McQuade R., Stojanovska V., De Leiris J., Nurgali K. (2018). PARP inhibition in platinum-based chemotherapy: Chemopotentiation and neuroprotection. Pharmacol. Res..

[45] Zhang T., Berrocal J.G., Yao J., Dumond M.E., Krishnakumar R., Ruhl D.D., Ryu K.W., Gamble M.J., Kraus W.L. (2012). Regulation of Poly(ADP-ribose) Polymerase-1-dependent Gene Expression through Promoter-directed Recruitment of a Nuclear NAD+ Synthase. J. Boil. Chem..

[46] Berger F., Lau C., Ziegler M. (2007). Regulation of poly(ADP-ribose) polymerase 1 activity by the phosphorylation state of the nuclear NAD biosynthetic enzyme NMN adenylyl transferase 1. Proc. Natl. Acad. Sci. USA.

[47] Park H.J., Bae J.S., Kim K.M., Moon Y.J., Park S.-H., Ha S.H., Hussein U.K., Zhang Z., Park H.S., Park B.-H. (2018). The PARP inhibitor olaparib potentiates the effect of the DNA damaging agent doxorubicin in osteosarcoma. J. Exp. Clin. Cancer Res..

[48] Li S., Cui Z., Meng X. (2016). Knockdown of PARP-1 Inhibits Proliferation and ERK Signals, Increasing Drug Sensitivity in Osteosarcoma U2OS Cells. Oncol. Res. Featur. Preclin. Clin. Cancer Ther..

[49] Engert F., Kovac M., Baumhoer D., Nathrath M., Fulda S. (2016). Osteosarcoma cells with genetic signatures of BRCAness are susceptible to the PARP inhibitor talazoparib alone or in combination with chemotherapeutics. Oncotarget.

[50] Chen K., Zhu C., Cai M., Fu N., Cheng B., Cai Z., Li G., Liu J. (2014). Integrative metabolome and transcriptome profiling reveals discordant glycolysis process between osteosarcoma and normal osteoblastic cells. J. Cancer Res. Clin. Oncol..

[51] Zhang H., Guo X., Feng X., Wang T., Hu Z., Que X., Tian Q., Zhu T., Guo G., Huang W. (2016). MiRNA-543 promotes osteosarcoma cell proliferation and glycolysis by partially suppressing PRMT9 and stabilizing HIF-1α protein. Oncotarget.

[52] Xiao Q., Wei Z., Li Y., Zhou X., Chen J., Wang T., Shao G., Zhang M., Zhang Z. (2018). miR-186 functions as a tumor suppressor in osteosarcoma cells by suppressing the malignant phenotype and aerobic glycolysis. Oncol. Rep..

[53] Song Y.-D., Li D.-D., Guan Y., Wang Y.-L., Zheng J. (2017). miR-214 modulates cisplatin sensitivity of osteosarcoma cells through regulation of anaerobic glycolysis. Cell. Mol. Boil..

[54] Han X., Yang Y., Sun Y., Qin L., Yang Y. (2018). LncRNA TUG1 affects cell viability by regulating glycolysis in osteosarcoma cells. Gene.

[55] Mizushima E., Tsukahara T., Emori M., Murata K., Akamatsu A., Shibayama Y., Hamada S., Watanabe Y., Kaya M., Hirohashi Y. (2019). Osteosarcoma-initiating cells show high aerobic glycolysis and attenuation of oxidative phosphorylation mediated by LIN28B. Cancer Sci..

[56] Gorospe M., De Cabo R. (2008). AsSIRTing the DNA damage response. Trends Cell Boil..

[57] Feige J.N., Auwerx J. (2007). Transcriptional coregulators in the control of energy homeostasis. Trends Cell Boil..

[58] Abdellatif M. (2012). Sirtuins and pyridine nucleotides. Circ. Res..

[59] Hu J., Deng F., Hu X., Zhang W., Zeng X., Tian X. (2018). Histone deacetylase SIRT6 regulates chemosensitivity in liver cancer cells via modulation of FOXO3 activity. Oncol. Rep..

[60] Sociali G., Galeno L., Parenti M.D., Grozio A., Bauer I., Passalacqua M., Boero S., Donadini A., Millo E., Bellotti M. (2015). Quinazolinedione SIRT6 inhibitors sensitize cancer cells to chemotherapeutics. Eur. J. Med. Chem..

[61] Zhao J., Wozniak A., Adams A., Cox J., Vittal A., Voss J., Bridges B., Weinman S.A., Li Z. (2019). SIRT7 regulates hepatocellular carcinoma response to therapy by altering the p53-dependent cell death pathway. J. Exp. Clin. Cancer Res..

[62] Jiang Y., Han Z., Wang Y., Hao W. (2018). Depletion of SIRT7 sensitizes human non-small cell lung cancer cells to gemcitabine therapy by inhibiting autophagy. Biochem. Biophys. Res. Commun..

[63] Zhang T., Berrocal J.G., Frizzell K.M., Gamble M.J., Dumond M.E., Krishnakumar R., Yang T., Sauve A.A., Kraus W.L. (2009). Enzymes in the NAD+ Salvage Pathway Regulate SIRT1 Activity at Target Gene Promoters*. J. Boil. Chem..

[64] Lakatos P., Hegedűs C., Ayestarán N.S., Juarranz Á., Kövér K.E., Szabo E., Virág L. (2016). The PARP inhibitor PJ-34 sensitizes cells to UVA-induced phototoxicity by a PARP independent mechanism. Mutat. Res. Mol. Mech. Mutagen..

[65] Hegedűs C., Lakatos P., Oláh G., Tóth B.I., Gergely S., Szabo E., Bíró T., Szabó C., Virág L. (2008). Protein kinase C protects from DNA damage-induced necrotic cell death by inhibiting poly(ADP-ribose) polymerase-1. FEBS Lett..

[66] Regdon Z., Robaszkiewicz A., Kovács K., Rygielska Ż., Hegedűs C., Bodoor K., Szabó É., Virág L. (2019). LPS protects macrophages from AIF-independent parthanatos by downregulation of PARP1 expression, induction of SOD2 expression, and a metabolic shift to aerobic glycolysis. Free. Radic. Boil. Med..

[67] Hegedűs C., Lakatos P., Kiss-Szikszai A., Patonay T., Gergely S., Gregus A., Bai P., Haskó G., Szabo E., Virág L. (2013). Cytoprotective dibenzoylmethane derivatives protect cells from oxidative stress-induced necrotic cell death. Pharmacol. Res..

[68] Gergely S., Hegedűs C., Lakatos P., Kovács K., Gáspár R., Csont T., Virág L. (2015). High Throughput Screening Identifies a Novel Compound Protecting Cardiomyocytes from Doxorubicin-Induced Damage. Oxidative Med. Cell. Longev..

[69] Erdélyi K., Bai P., Kovács I., Szabó É., Mocsár G., Kakuk A., Szabó C., Gergely P., Virág L. (2009). Dual role of poly(ADP-ribose) glycohydrolase in the regulation of cell death in oxidatively stressed A549 cells. FASEB J..

[70] Aladdin A., Király R., Boto P., Regdon Z., Tar K. (2019). Juvenile Huntington’s Disease Skin Fibroblasts Respond with Elevated Parkin Level and Increased Proteasome Activity as a Potential Mechanism to Counterbalance the Pathological Consequences of Mutant Huntingtin Protein. Int. J. Mol. Sci..

[71] Kovács P., Csonka T., Kovács T., Sári Z., Ujlaki G., Adrienn S., Karányi Z., Szeőcs D., Hegedűs C., Uray K. (2019). Lithocholic Acid, a Metabolite of the Microbiome, Increases Oxidative Stress in Breast Cancer. Cancers.

