# Supplementary material for: Targeting Nuclear NAD+ Synthesis Inhibits DNA Repair, Impairs Metabolic Adaptation and Increases Chemosensitivity of U-2OS Osteosarcoma Cells
Source: Cancers (Basel). 2020 May 7;12(5):1180. doi: 10.3390/cancers12051180 (PMC7281559; doi:10.3390/cancers12051180)
Supplement: Supplementary file 1 [file cancers-12-01180-s001.zip › Supplementary Material0506.docx]

Supplementary Material: Targeting Nuclear NAD^+^ Synthesis Inhibits DNA Repair, Impairs Metabolic Adaptation and Increases Chemosensitivity of U-2OS Osteosarcoma Cells

Alexandra Kiss, Arnold Péter Ráduly, Zsolt Regdon, Zsuzsanna Polgár, Szabolcs Tarapcsák, Isotta Sturniolo, Tarek El‐Hamoly, László Virág and Csaba Hegedűs


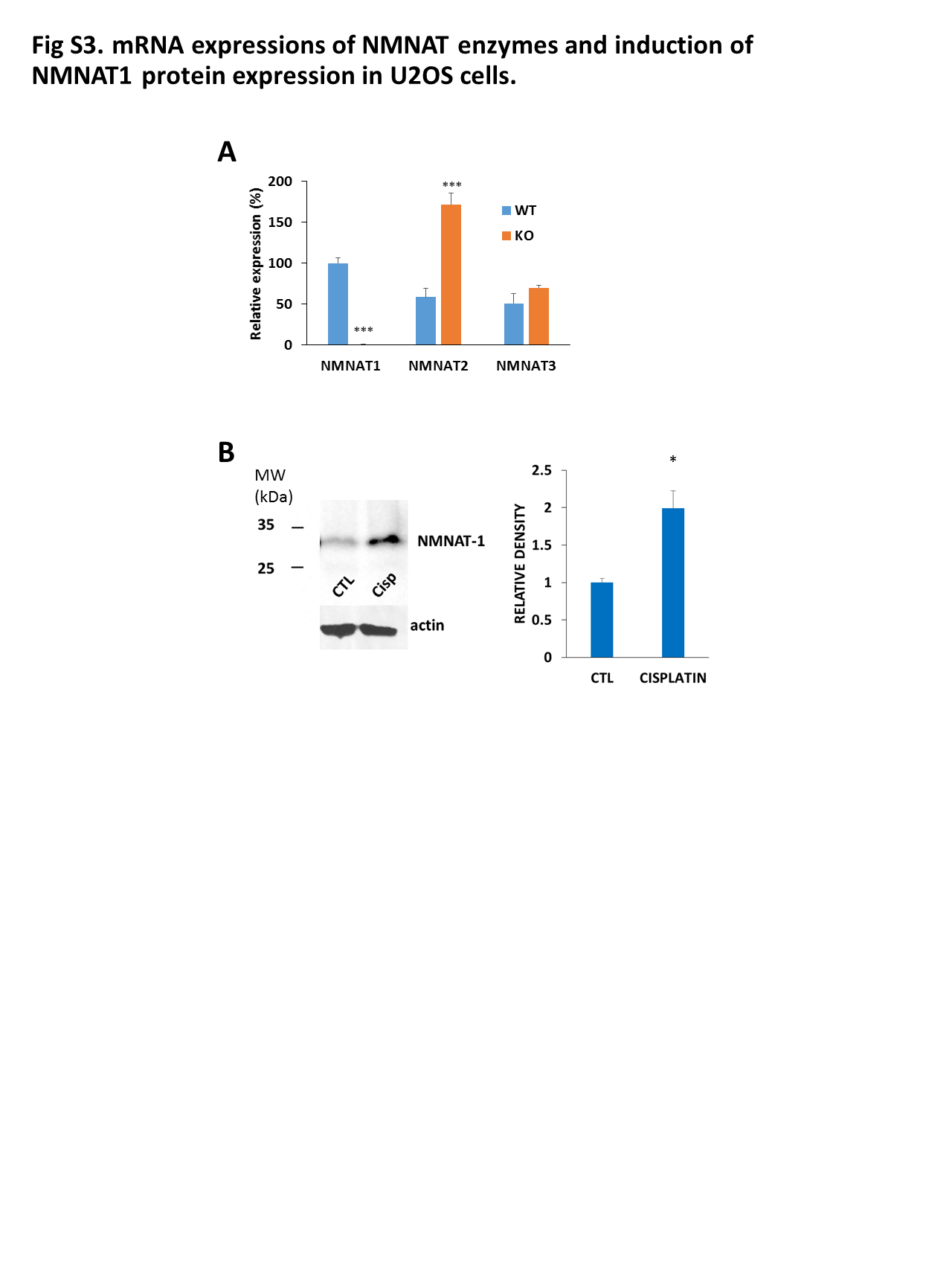


**Figure S1.** mRNA expressions of NMNAT enzymes and induction of NMNAT1 protein expression in U-2OS cells. The expression of NMNAT1, NMNAT2 and NMNAT3 mRNA was determined in WT and NMNAT1 KO U-2OS cells lines. Bars marked with asterisks are significantly different from the expression of the same NMNAT gene in WT cells. (Student–Newman–Keuls method; *** *p* < 0.05) (**A**). NMNAT1 protein expression in the U-2OS cell line was detected 24 hours after cisplatin (5 μg/mL) treatment. Bars marked with asterisks are significantly different from the control (Student’s *t* test; * *p* < 0.05) (**B**). Full WB image can be found in Supplementary Material. Data plotted are means ± SEM (*n* = 3).


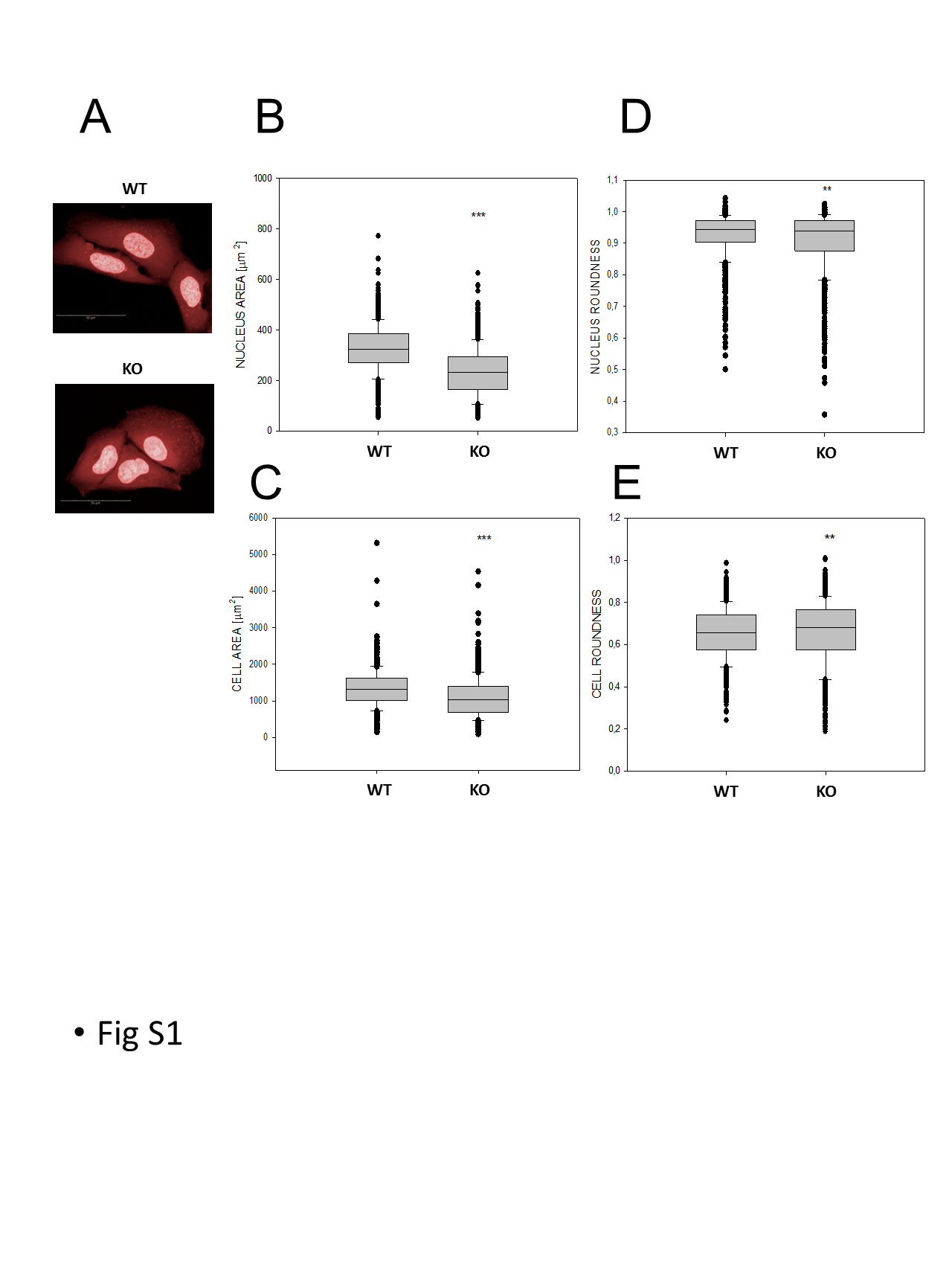


**Figure S2.** Cell morphology analysis. Wild type and NMNAT1 KO U-2OS cells were stained with DRAQ5 dye. Images were taken with Opera Phenix High Content Analyzer (**A**). Morphological phenotyping was conducted to reveal differences between WT and KO cells. Nuclear area (**B**), cell area (**C**), nuclear roundness (**D**), and cell roundness (**E**) were measured. Boxes marked with asterisks are significantly different from the wild type sample (Tukey test; ** *p* < 0.01, *** *p* < 0.001,). Data plotted are means ± SEM (*n* = 3).


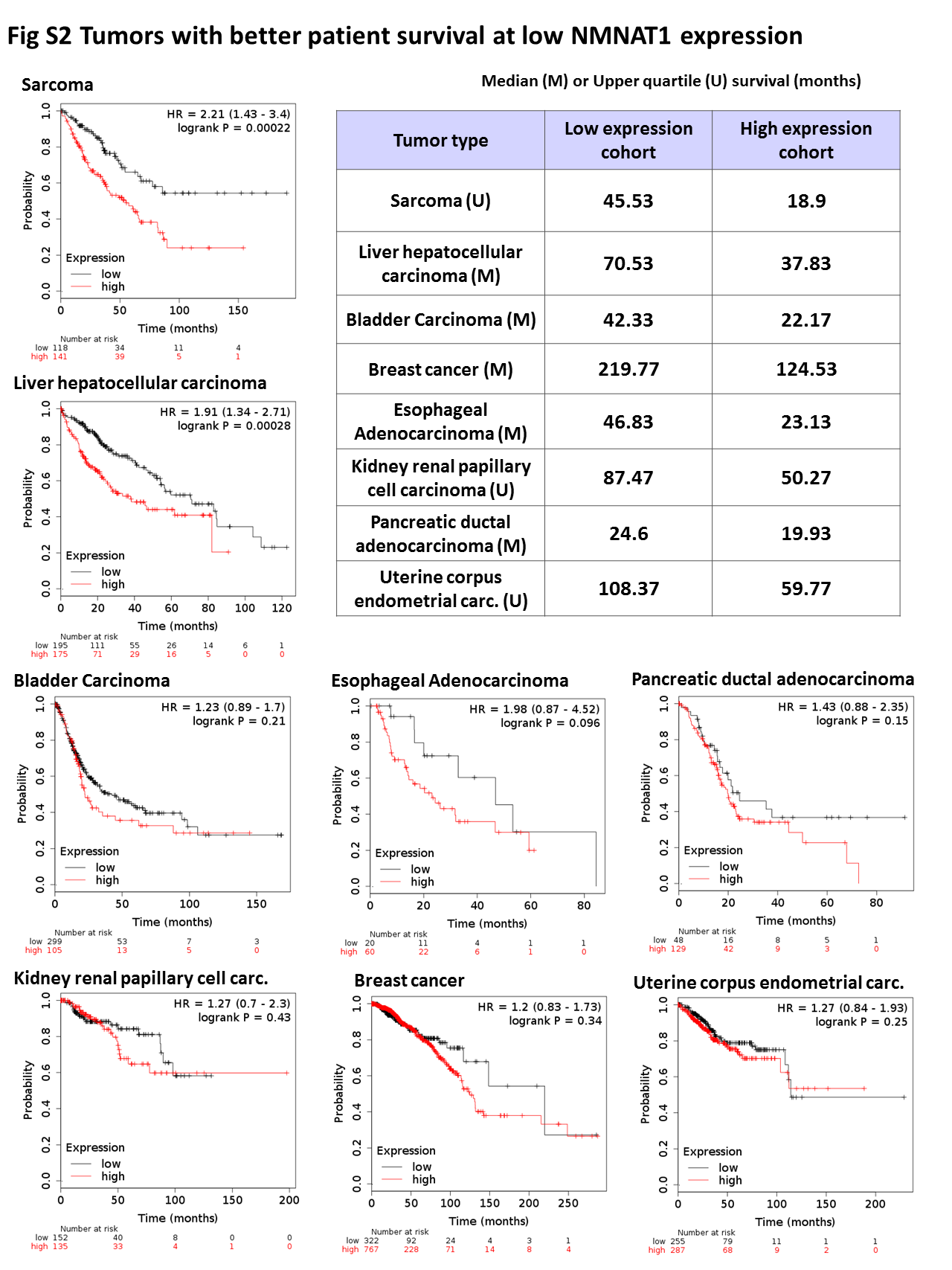


**Figure S3.** Tumors with better patient survival at low NMNAT1 expression. RNAsec data from kmplot.com were used to construct the graphs. The probabilities of survival are presented on the graph as a function of time (months). The median (M) or upper quartile (U) survival rates of the low and high NMNAT1 expression cohorts are compared.

**Table S1.** Antibodies used for western blotting.

| **Antibody Specific for** | **Host Species** | **Cat#** | **Supplier** | **Dilution** |
| --- | --- | --- | --- | --- |
| poly(ADP-ribose) | mouse | N/A | produced in house (10H hybridoma) | 1:500 |
| NMNAT1 | rabbit | sc-271557 | Santacruz  (Dallas, TX, USA) | 1:1000 |
| human actin | mouse | sc-47778 | Santacruz  (Dallas, TX, USA) | 1:20000 |
| anti-mouse IgG (HRP) | horse | 7076S | Cell Signaling  (Danvers, MA, USA) | 1:3000 |
| anti rabbit IgG (HRP) | goat | 7074S | Cell Signaling  (Danvers, MA, USA) | 1:1500 |

**Table S2.** Antibodies and dyes used for High Content Analysis.

| **Antibody Specific for** | **Host Species** | **Cat#** | **Supplier** | **Dilution/Concentration** |
| --- | --- | --- | --- | --- |
| anti-phospho-H2AX | mouse | 4418-APC-100 | Trevigen  (Gaithersburg, MD, USA) | 1:1000 |
| anti-rabbit, Alexa Fluor 488 | *goat* | A1108 | Invitrogen  *(*Waltham, MA USA) | 1:2000 |
| Annexin V-FITC | - | 640945 | Biolegend  (San Diego, CA, USA) | 1:100 |
| CellEvent™ Caspase-3/7 Green Detection Reagent | - | C10423 | Thermo Fischer  (Waltham, MA USA)) | 7 µM |
| DAPI | - | #013606 | Molecular Probes  (Eugene, Oregon, USA) | 0.5 μg/mL |
| DRAQ5 | - | 65-0880-92 | ThermoFischer/eBioscience  (Waltham, MA USA) | 2.5 μM |
| Hoechst | - | 33342 | Molecular Probes  (Eugene, OR, USA) | 1:1000 |

**Table S3.** Sequences of primers, used in quantitative PCR experiments.

| **Primers** | **Fw** | **Rw** |
| --- | --- | --- |
| ***hNMNAT1*** | *AAAGGCCTGGAAGGAAGAGG* | *CCCATAGTTGGCCACGATTT* |
| ***hNMNAT2*** | *CATATTTCCTGGTGGCATCT* | *CTGGTCCTGCCTATGTGGTT* |
| ***hNMNAT3*** | *GCGCACATCCAGGAAATAGT* | *TGGCACTGATCTCATTCTGC* |
| ***hATP5B*** | *TCACCCAGGCTGGTTCAGA* | *AGTGGCCAGGGTAGGCTGAT* |
| ***hHSP60*** | *CACCGTAAGCCTTTGGTCAT* | *CTTGACTGCCACAACCTGAA* |
| ***hPKM2*** | *ATGAGTACCATGCGGAGACC* | *TGTCTAGAGCCACAGCAACG* |
| ***h36B4*** | *CCATTGAAATCCTGAGTGATGTG* | *GTCGAACACCTGCTGGATGAC* |
| ***hcyclophilin*** | *GTCTCCTTTGAGCTGTTTGCAGAC* | *CTTGCCACCAGTGCCATTATG* |
